# Lived experiences of diabetes self-management in North Shoa, Ethiopia: *A phenomenological inquiry*

**DOI:** 10.1371/journal.pone.0316505

**Published:** 2026-06-30

**Authors:** Akine Eshete, Abera Lambebo, Lemma Getacher, Tewodros Kifleyohans, Yibeltal Assefa, Hendry Van Der Heever, David D. Mphuthi

**Affiliations:** 1 Department of Public Health, Debre Berhan University, Debre Berhan, Ethiopia; 2 Department of Health Studies, School of Social Sciences, University of South Africa, Pretoria, South Africa; 3 Department of Surgery, Debre Berhan University, Debre Berhan, Ethiopia; 4 School of Public Health, The University of Queensland, Brisbane, Australia; National Research Centre, EGYPT

## Abstract

**Background:**

Despite government efforts, diabetes self-management remains inadequate for many patients due to complex barriers. Understanding the lived experiences and barriers to self-care practices is essential for facilitating behavior change and achieving personal health goals for improved diabetes management. Therefore, this study aimed to explore the lived experiences and barriers faced by individuals living with diabetes in adhering to their self-care practices.

**Methods:**

This phenomenological study was carried out in the North Shoa Zone from 1 to 30 July 2024. The study consisted of a total of 25 participants, 20 diabetic patients and five healthcare informants from four districts. The participants were selected using maximum variation sampling method, considering factors such as age, sex, marital status, occupation, and type of diabetes. A pretested interview guide was used to gather the data, which were then recorded, transcribed, and analyzed with ATLAS.Ti software. A thematic framework was applied to identify key codes, subthemes, and main themes associated with diabetes self-care and the barriers to its practice.

**Results:**

Diabetes self-management behaviors were found to be insufficient, mainly due to barriers at the individual, interpersonal, and community levels. Despite high adherence to prescribed medications intake among most patients, many still have inadequate practices in self-blood glucose monitoring, diet, regular physical activity, and foot care. Key barriers included limited knowledge of self-care, socioeconomic constraints, lack of guidance, low motivation, stress, limited social support, cultural influences, and poor access to resources.

**Conclusions:**

Diabetes self-care was inadequate due to several challenging barriers. To improve these practices, it is crucial to integrate behavioral change interventions, provide mental health support, implement stress management strategies, and foster community partnerships to address barriers comprehensively at all levels.

## Background

Diabetes mellitus (DM) is a rising global health threat, affecting 10.5% of adults in 2021 and projected to reach 12.2% by 2045, with 80.6% of cases occurring in middle-income countries [1,2]. Diabetes poses a major health challenge in Ethiopia, affecting 1.92 million adults, with cases projected to rise to 2.74 million by 2030 and 4.76 million by 2045 [1,3], requiring continuous behavioral adjustments for effective self-care [4–7]. People with diabetes should be encouraged to adopt healthy lifestyle habits to control blood sugar, prevent complications, and enhance overall well-being [4–6].

Effective diabetes management requires lifelong lifestyle changes, including diet, exercise, glucose monitoring, and medication adherence, supported by adequate knowledge and skills [7–9]. Despite advanced interventions, patients in resource-limited settings continue to face significant challenges in maintaining effective self-care behaviors [9–13].

Inadequate self-care and limited knowledge contribute to diabetes-related morbidity and mortality, with poor adherence and lifestyle modifications being major obstacles [14]. In Ethiopia, diabetes self-management remains a critical public health challenge, despite ongoing government efforts [10–13,15]. Research findings in Ethiopia indicates that over 60% of patients have insufficient self-care management, especially regarding diet, exercise, foot care, and glucose monitoring [16–18]. In addition, recent studies report 54% [19] to 61% [20] of patients demonstrate inadequate self-care practices, particularly in terms of foot care, dietary habits, exercise, and self-monitoring of blood sugar levels [21].

Various studies highlight key barriers to effective diabetes self-management, including limited knowledge, low motivation, weak social support, financial constraints, high treatment costs, and limited access to healthcare [22–24]. These challenges highlight the urgent need for comprehensive evidence on the lived experiences and barriers patients face, especially in resource-limited countries [24,25]. Understanding how personal, social, and systemic factors influence diabetes management emphasizes the need for tailored support approaches [26].

In Ethiopia, qualitative research on lived experiences with diabetes self-management is limited and inconsistent [27–29]. However, barriers to effective self-care arise from a range of individual, psychological, social, and economic factors. [21,22,29–31]. Improving diabetes self-management demands a comprehensive understanding of the lived experiences and challenges individuals encounter [7]. This study used a phenomenological approach to explore patients’ lived experiences and barriers to diabetes self-management in the North Shoa Zone. It aims to inform personalized care plans and improve management strategies [26].

## Method of study

### Study area and design

This qualitative phenomenological study was conducted in the northern Shoa Zone, Amhara region, from 1 to 30, July 2024. This zone, which borders Oromia, South Wollo, and Afar, includes 32 districts, with Debre Berhan as the capital city, located 130 km northeast of Addis Ababa [32]. A phenomenological approach was employed to investigate the lived experiences of individuals with diabetes and the barriers to effective self-management. It focuses on understanding individuals’ personal accounts of their situations, feelings, and challenges. Through systematic analysis, this approach aims to identify the core meaning of self-management practices [33].

### Population and sampling methods

The study involved 20 diabetic patients and five key healthcare informants from four districts in the Zone, with five participants interviewed from each district. The number of interviews was guided by the principle of data saturation, concluding when no new information or insights were gained, in line with existing research recommendations [34,35]. All eligible diabetic patients who were aged 18 years and above, resided in the zone, and agreed to participate were included in the study. Patients who were severely ill and unable to communicate during the data collection process were not included in the study. Participants were selected using maximum variation sampling, considering age, sex, marital status, occupation, and type of diabetes, to capture a wide range of self-care experiences and challenges.

### Interview process and data collection method

Data were collected by trained data collectors with support from two academic lecturers from Debre Berhan University, and the researchers closely supervised the entire data collection process. All interviewed data were recorded using audio equipment. The interview guide was developed through comprehensive literature and consultations with experts in the field [36]. The interview guides were first developed in English, translated into Amharic, and subsequently reviewed and validated by subject matter experts.

The guiding tool focused on exploring individuals’ experiences in diabetes management, particularly diet and nutrition, physical activity, medication adherence, blood glucose monitoring, and foot care. It also examined the challenges they faced in these areas and how these challenges affected their ability to manage their condition effectively. In addition, it included prompts to explore barriers to self-management and questions seeking participants’ suggestions for improving their practices.

The interview guide was pretested in a comparable healthcare facility with a similar patient population and service context to the study setting, but outside the actual study sites, to ensure its clarity, relevance, and appropriateness for both participants and interviewers. Consent was obtained from each participant before the interviews, which were scheduled at convenient times and locations and lasted 30–40 minutes.

### Trustworthiness of the study

Qualitative researchers focus on trustworthiness, which includes credibility (internal validity), dependability (reliability), transferability (external validity), and confirmability (presentation) [37–41]. Credibility was ensured by employing triangulation, achieving data saturation, and rigorously testing interview guides to capture participants’ perspectives accurately. This involved using of various data sources and carefully reviewing of transcriptions and coding. Dependability was maintained through careful documentation, regular error checks, and a clear explanation of the study procedures. Confirmability was addressed by minimizing biases, validating results with feedback, using investigator triangulation, and preserving audio recordings for transparency. The findings were also peer reviewed before finalization. Finally, transferability was supported by diverse sampling methods and comprehensive descriptions of the study context, participants, and data collection procedures.

### Researcher reflexivity statement

In this study, we reflected on our assumptions, biases, and experiences as diabetes researchers to maintain objectivity and minimize our influence on data collection, analysis, and interpretation. The research team critically engaged with the data collected from participants and relevant literature. The multidisciplinary expertise and prior experience also shape our understanding of the research context, particularly patients’ experiences and perspectives on self-care practices. The research team did not directly interact with participants during interviews prior to the study. Despite our efforts, personal biases may have influenced the research. By recognizing this, we seek to improve transparency and promote a critical assessment of the findings.

### Data management and analysis method

The collected data were transcribed verbatim in Microsoft Word and analyzed via ATLAS.ti v25 software. A thematic framework approach was used to identify key themes and explore participants’ perspectives on diabetes self-care and challenges [42,43].

**Fig 1:**
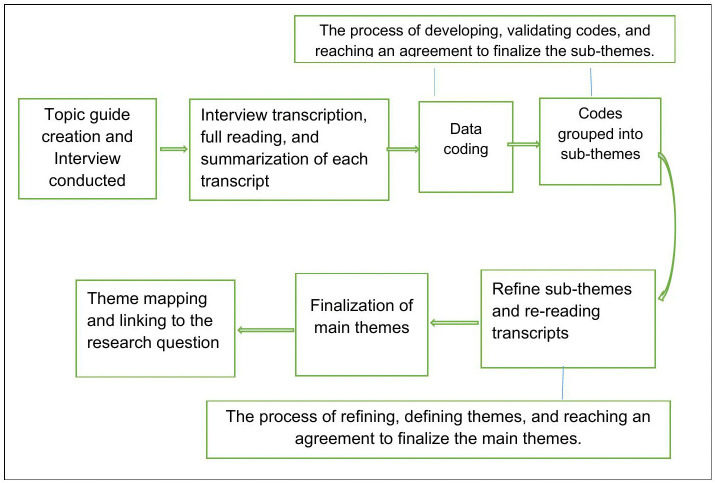
Data analysis process including the mapping of codes, subthemes, and main themes related to diabetes self-management and associated barriers. This thematic framework illustrates how raw data were categorized and abstracted into meaningful themes during qualitative analysis.

The data analysis process involved creating codes, subthemes, and main themes related to diabetes self-management and its barriers. The transcripts were repeatedly reviewed to become familiar with the data and to identify key concepts. Initial coding captured specific aspects of participants’ experiences. These codes were then grouped into subthemes and further organized into main themes, forming a structured framework for understanding diabetes self-management and related challenges (Fig 1).

Diabetic self-care experiences were grouped under the main theme of adherence to self-care practices. The initial analysis identified five main subthemes and 18 categories or codes. The subthemes included dietary practices, regular physical activity, anti-diabetic medication adherence, self-monitoring of blood glucose levels, and foot care. Barriers to diabetes self-care included inadequate self-care knowledge, psychological challenges, socioeconomic issues, lack of support, provider-related obstacles, cultural factors, and restricted access to resources.

Finally, the themes were reviewed to ensure that they addressed the research questions. Narrative information was organized by emerging topics and concepts, and direct quotes from participants were included to reflect their views [43].

### Ethics approval and consent to participate

Ethical approval was granted by the Institutional Review Board at Asrat Woldeyes Health Science Campus, Debre Berhan University, and the Research Ethics Review Committee at the College of Human Sciences, University of South Africa (Approval No. 19833555_CREC_CHS_2024). Written informed consent was obtained from all participants. To ensure confidentiality, personal identifiers were removed, and anonymous codes were used during both data collection and analysis. Participants’ rights to withdraw or decline to answer any questions were fully respected, and strict data protection protocols were maintained throughout the study.

## Results

### Demographic and socioeconomic characteristics of the study participants

#### Sociodemographic profiles of patients with diabetes.

A total of 20 diabetic patients were included in the in-depth interviews. Of these, 60% were men, 60% were middle-aged, and 60% lived in urban areas. Additionally, 45% of participants were unsure of their specific type of diabetes (Table 1).

**Table 1:** Sociodemographic profiles of patients with diabetes in the northern Shoa zone, Amhara region, Ethiopia, 2024.

Variables	Frequency n (%)
**Sex of the respondent**	
Male	12 (60%)
Female	8 (40%)
**Age of the respondent**	
Young age (25–44)	6 (30%)
Middle age (44–60)	12 (60%)
Elderly age (>60)	2 (10%)
**Place of residence**	
Urban	8 (40%)
Rural	12 (60%)
**The employment status of the respondents**	
Government employee	6 (30%)
Private/merchant	1 (5%)
Farmer	3 (15%)
Housewife	5 (25%)
Daily worker and self-worker	5 (25%)
**Type of diabetes**	
Type one diabetes	4 (20%)
Type two diabetes	7 (35%)
I don’t know the type of diabetes	9(45%)

#### Sociodemographic characteristics of the health care provider.

Key informant interviews were conducted with five healthcare providers. Among theme three were men, mostly were aged 25–44 years, and two were internal medicine residents. All providers worked in chronic care follow-up clinics, with an average of 2.2 years of experience (Table 2).

**Table 2:** Sociodemographic characteristics of health care providers in the North Shoa Zone, Amhara Region, Ethiopia, 2024.

Variables	Frequency n (%)
**Sex of the respondent**	
Male	3 (60%)
Female	2 (40%)
**Age of the respondent**	
The mean age of the respondent	30.5 + 6.2
Young age (25–44)	3 (60%)
Middle age (44–60)	2 (40%)
**Profession of the respondents**	
Physician	4 (80%)
Nurse	1 (20%)
**Working experience of the respondents**	
Mean working experience	2.2 + 1.32
Less than two year	2 (40)
Greater than two year	3 (60)

### Adherence to self-management behaviors

Diabetic self-management behaviors were summarized under the main theme of adherence to recommended self-management behavior. This main theme was further categorize into five subthemes including dietary habits, regular physical activity, adherence to anti-diabetic medications, self-monitoring of blood glucose, and foot care (Table 3).

**Table 3:** Summary of self-management behaviors among patients with diabetes in the North Shoa Zone, Amhara Region, Ethiopia, 2024.

*Main theme*	*Subtheme/categories*	Selected coded statement
Adherence to self-management behaviors	Regular physical exercise	Exercised 2–3 days a week
		Walking is the only way to exercise.
		Doing activities related to daily work
		Do not do any physical exercise in their life including walking.
		No consideration of intensity-moderate activities
	Dietary practice	Didn’t have a healthy eating plan in place
		Despite lacking a weekly plan, they mainly eat vegetables, fruits and other recommended foods.
		Did not consume enough fruits and vegetables, evenly space carbohydrates, and include foods with low glycemic index.
		Eating what they got without making specific choices
	Regularly taking anti-diabetic medication.	Regularly taking anti-diabetic medication.
		Skipping medication or injections during fasting
		Stopping medication due to instability in their area
	Self-blood glucose monitoring	Did not regularly check their blood glucose levels
		Lacks a glucometer and is unfamiliar with its use
	Foot care practice	Wash and inspect their feet every day.
		Did not regularly check their feet every day
		Inconsistent health checks and washing

#### Theme one: Adherence to diabetic self-management behaviors.

Diabetes self-management remains a significant challenge for most respondents, with inconsistent adherence. Most participants did not have proper diabetic self-care practice due to their focus on daily work-related activities. Participants reported that persistent social challenges, along with the nature of the disease, continue to hinder their adherence to recommended self-care. Healthcare providers noted that despite discussing the importance of self-care practices with patients, many do not yet follow recommended behaviors, especially in terms of physical activity and dietary habits.

*“…Life is hard now. What kind of self-care practices are there? I do not have any self-care practices. Nothing I do every day…”(A 78-year-old man with T1DM and 70-year-old man with T2DM)*

*‘...Adherence to recommended self-care behaviors is inadequate, particularly concerning dietary habits, physical activity, foot care, and self-glucose monitoring. Patients often eat whatever is available and have limited access to exercise facilities.” (Male, medical doctor)*

Some participants mentioned that when first diagnosed with diabetes, they did not follow the recommended self-care practices. Over time, some adopted certain practices, believing that they were crucial for survival. As they have aged, they intend to follow these practices to support their current health and survival.

*“I did not follow the recommendations when first diagnosed, but after my blood sugar levels increased, I started taking action, although my effort was still limited.” (55-year-old man with T2DM)*

*“... As people age, they follow these practices to live longer. Since I am still young and not worried about dying, I have not strictly followed the recommended self-care practices.” (22-year-old man with T1DM)*

**Subtheme one**: **adherence to regular physical exercise.** Almost all of the respondents reported that regular physical exercise is not part of their daily activities. Only a few participants participate in exercise 2–3 days a week, and the most common activities are walking (including brisk walking and hiking), jumping rope, and gym use.

Although most of the participants reported walking a main type of of physical activity, they did not participate in other types of exercise. Most of the participants did not engage in moderate-intensity activities because, as they were occupied with other responsibilities and had limited understanding of various type of exercise.

*“I don’t engage in any recommended physical exercise apart from household chores. Occasionally, I walk, but my physical activity is mostly limited to moving around the house.” (54-year-old housewives, 60-year-old housewife and 55-year-old man with T2DM)*

*“… I engage in physical labor related to agricultural work but do not follow the specific exercises recommended for diabetic patients.” (32-year-old farmer T2DM; and 28-year-old farmer)*

**Subtheme two: adherence to dietary practices.** Maintaining healthy eating practices is a major challenge for most respondents. However, a few participants effectively managed their diet, consuming mainly vegetables, fruits, and other recommended foods, despite lacking a weekly meal plan.

*“I avoid foods that raise my sugar levels and stick to eating vegetables, fruits, and other recommended foods.” (50-year-old man with T1DM)*

*“... Since my diagnosis, I have been avoiding forbidden foods and adhering to recommended foods for diabetic patients.” (25-year-old man)*

Most participants lack a healthy eating plan, often consuming whatever food is available without following a balanced diet that includes fruits, vegetables, balanced carbohydrates, and low glycemic index foods. Most participants noted that not following diabetes-specific meal plans and eating without careful dietary choices are major challenges.

*‘... My sugar level is rising because I eat whatever food is available. I consume milk, meat, butter, foods high in sugar, and various other foods.” (60-year-old housewives)*

*“I avoid sweet and fatty foods, but I don’t follow the recommended diet because I cannot find the recommended foods.” (54-year-old man with T2DM)*

Diabetic patients and healthcare providers said injera is the main daily consumed food, with little focus on a balanced diet. Patients follow regular meal programs similar to those of non-diabetics. Many people find it hard to prepare separate meals. Ethiopian food is often high in salt and unhealthy oils, making it less suitable for people with diabetes. Social events also significantly influence and encourage unhealthy eating habits.

*‘...Life is not what I expected. I eat what is available rather than following a specific eating plan or diet.” (70-year-old man with T2D, p#8 and 54-year-old man with T2DM)*

*‘...When I attended social events such as weddings, holidays, and religious gatherings, I often made unhealthy food choices and consumed local soft drinks and alcohol.’ (28-year-old farmer)*

**Subtheme three: regularly taking antidiabetic medication.** Most of the respondents believed that taking anti-diabetic medication is essential for survival and reported being more consistent with their medication than with other self-care practices. However, some participants reported problems with their medication practices, such as skipping injections during fasting periods and discontinuing medication intake due to shortages drug caused by local instability.

*“I stopped my injections when my medication ran out and I could not go to the hospital because of instability in my area.” (23-year-old woman with T1DM)*

**Subtheme four: regular self-blood glucose monitoring.** Despite its importance, blood glucose testing is the least familiar self-care practice among most of the respondents. Many participants reported that they did not regularly monitor their blood glucose levels and only checked them during scheduled follow-up visits at healthcare facilities. Almost all the respondents recognized the importance of blood glucose testing but they lack a glucometer and do not know how to use it.

*“--- It is crucial to check my blood glucose when I feel unwell, but I do not have a glucometer and do not know how to use one.” (45-year-old woman with T2DM)*

**Subtheme five: foot care practice.** Foot care was the least practiced self-care behavior among respondents, with only a few consistently washing and inspecting their feet daily. Almost all the participants did not perform the recommended foot care practices, including washing with warm water, drying, and providing proper care. Although many participants do not perform foot care daily, some of theme sometimes wash and inspect their feet.

*“I don’t check my feet every day, but I try to keep them safe and healthy.” (22-year-old man with T2DM)*

*“I didn’t know that diabetics needed to regularly inspect, wash, and dry their feet. I am not aware of the proper methods, so I have not done it.” (55-year-old man with T2DM)*

### Barriers to diabetes self-management practices

**Fig 2:**
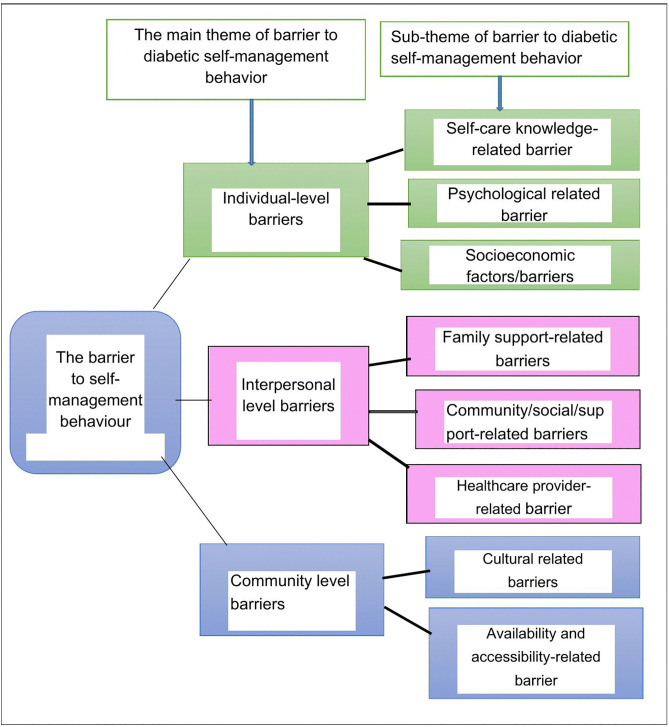
Summary of key barriers to diabetes self-management behaviors among diabetic patients. The figure highlights individual and contextual factors.

Challenges and barriers to diabetes self-management were categorized, with key barriers including a lack of knowledge, inadequate guidance, low motivation, stress, insufficient support, socioeconomic difficulties, cultural factors, and limited access to resources (Fig 2).

The main barrier to self-management of diabetes is a lack of fundamental knowledge about essential self-care practices. This lack of understanding has contributed to inadequate adherence to dietary practice, regular physical activity, blood glucose monitoring, and proper foot care.

“... I’m *not familiar with different types of exercise beyond walking, as my doctor advised. I also lack information on recommended foods for diabetics and I eat whatever is available.” (45-year-old woman with T2DM and a 56-year-old housewife)*

Many respondents faced psychological barriers to diabetic self-management, including low motivation, stress, and emotional distress. An unstable lifestyle and lack of focus make it difficult to follow recommended self-care tasks. Healthcare providers reported that low motivation is linked to poor understanding of self-care. In addition, participants said that pain and the severity of their condition strongly influence their adherence.

*“... I often neglect self-care owing to impatience and anger, resulting in frequent illness and high blood sugar. When I am stressed and hopeless, I tend to eat whatever is available.” (60-year-old housewife and 23-year-old woman with T1DM)*

Financial limits were a major barrier for many participants, affecting their ability to make dietary changes, engage in physical activity, monitor glucose, and perform other self-care activities. Limited access to essential resources such as healthy food, medications, glucometers, educational materials, and exercise facilities significantly affects the management of diabetes.

*“... With my low income, I cannot afford a healthy diet or a glucometer. How can I manage self-care with these financial constraints?” (57-year-old farmer and 60-year-old woman)*

*“Some individuals with diabetes have lack of access to healthy food options at home, which leads them to consume whatever is available.” (Male, medical doctor)*

Many participants reported insufficient family support, making it difficult to prepare separate meals for themselves and their families, which led to unhealthy dietary practices. Because of limited family support, many people with diabetes have to manage their care on their own.

*“I understand the importance of a healthy diet, but I have to eat what is prepared at home. It is too challenging to make separate meals for myself and my family.” (56-year-old woman and 54-year-old man with T2DM)*

The participants noted that peer pressure and social events often result in poor self-care, especially unhealthy eating. Social events typically lack healthy food options, which leads to pressure to choose unhealthy foods. Additionally, some community members fear that starting medication early indicates that there is no cure and that diabetes will be a lifelong disease.

*“Some diabetes patients consumed forbidden foods at festivals and social events because healthy options were unavailable” (Medical doctor, male).*

*“At weddings, holidays, and religious events, the absence of suitable diabetic food often leads me to eat whatever is available.” (60-year-old housewives and 54-year-old men with T2DM)*

Many participants reported receiving inadequate and inconsistent self-care guidance from healthcare providers, leading to unclear management plans. Most patients said healthcare providers mainly focus on treatment rather than comprehensive self-care counseling because of high patient load and limited time. As a result, patients frequently lack adequate education on diet and exercise and may overlook physician recommendations.

*‘... Since 2005 CE, I have been advised to avoid sweets and walk, but I have not received sufficient guidance on self-care practices.’ (70-year-old man with T2DM)*

*“…When we seek treatment, we receive only basic care. Even if providers are busy, they should offer advice on nutrition, exercise, and self-care at least monthly. Physicians must prioritize education and raise awareness of self-care practices.” (55-year-old man with T2DM and* a *50-year-old man)*

## Discussion

### Adherence to diabetic self-management behaviors

This study explores diabetic self-management behaviors and key barriers. Diabetic self-management presents significant challenges for many patients, with barriers emerging at the individual, interpersonal, and community levels. The most commonly identified barriers included insufficient knowledge, economic constraints, inadequate guidance, psychological problems such as low motivation and stress, limited social support, cultural factors, and poor access to resources.

In this study, diabetic self-management behaviors were a major challenge for many patients, particularly with respect to self-monitoring of blood sugar, adhering to dietary practices, maintaining regular exercise, and practicing regular foot care. This study aligns with the findings of previous studies conducted in Ethiopia and other countries [10,16,17,19,20]. A phenomenological study in northeastern Ethiopia revealed that foot care, diet practices, regular exercise, and self-monitoring of blood sugar were neglected [21]. A systematic review in high-income Western countries reported low adherence to diabetes self-management, especially with respect to glucose monitoring, medication, diet, and physical activity [44]. This highlights the need to integrate self-care practices into daily schedules by addressing the specific challenges faced by individual patients.

In the present study, some participants adopted diabetic self-care practices after experiencing severe health problems, such as elevated blood sugar levels. This highlights the urgent need for proactive self-care strategies that address both practical and psychological barriers [45].

### Adherence to regular physical exercise

Despite the substantial body of evidence highlighting the advantages of regular physical activity for diabetes management, most study participants fail to engage in consistent regular exercise. Nearly all the participants reported a lack of regular exercise. Consistent findings in Ethiopian studies reported similar challenges in incorporating exercise into daily schedules [10,19,29,46–48]. This highlights a critical gap in integrating physical activity into diabetes management [49,50].

In this study, walking was the most common exercise, but participants rarely did other recommended physical activities. This finding aligns with both Ethiopian and global studies, where walking is commonly reported as the primary form of physical activity [20,51,52]. The study revealed that many participants did not participate in the moderate-intensity exercises recommended for diabetes management, a challenge that is consistent with previous research [10,46–48,53].

In this study, participants mistakenly believed that household work could substitute for regular exercise, which hindered their physical activity. This is consistent with findings from Pakistan [54]. Additionally, participants mistakenly perceive that exercise combined with medication was ineffective in lowering blood sugar. This misunderstanding leading them to stop exercising, despite substantial evidence emphasizing the benefits of regular exercise [55]. Similar misconceptions result in lower adherence to physical activity among diabetes patients [56].

### Adherence to dietary practices

In this study, many participants lacked a healthy diet plan and often made unhealthy food choices. Consistent findings were reported in studies in Ethiopia, showing that inadequate meal planning and unhealthy eating habits are common among individuals with diabetes [19,29,57–59]. This finding calls for practical, patient-centered strategies to improve dietary practices by strengthening nutrition education and awareness on how to develop appropriate meal plans based on available food options.

In the present study, some participants ate a diet rich in vegetables and fruits without formal planning, but most did not follow a balanced recommended diet and ate whatever was available. This is a common problem among people with diabetes in Ethiopia [19,29,57–60]. Addressing these dietary challenges is crucial to better managing diabetes, as supported by studies [61,62].

In this study, participants who ate whatever food was available experienced increased blood sugar levels. This finding is consistent with previous research, which indicates that inadequate meal planning and unhealthy eating negatively impact diabetes management, leading to worse outcomes [60]. Another study in Ethiopia demonstrated that insufficient diabetes management leads to increased blood glucose levels [63]. Improved diet guidance and access to healthy food are essential for better diabetes management and health outcomes [61,62].

### Regularly taking anti-diabetic medication

In this study, most participants regularly took their anti-diabetic medication, which is consistent with findings from Ethiopia, where patients generally demonstrate better adherence to their medication [29]. The study revealed that some participants had difficulties fasting and experienced medication shortages due to instability. Similar findings reported in prior studies, which highlight the need for clear guidelines for managing diabetes during fasting and improved medication access [49].

### Regular self-blood glucose monitoring

In this study, most respondents recognized the importance of blood glucose testing; however, they did not regularly monitor their levels and only had them checked at healthcare facilities during follow-up visits. In addition, limited access to glucometers and inadequate training and education hinder effective self-monitoring for most patients. This result is consistent with findings from studies conducted in Ethiopia and other countries, where self-monitoring is often neglected because of limited access to the glucometer and insufficient education [10,64,65]. A phenomenological study conducted in northeastern Ethiopia revealed that the blood sugar levels of the majority of patients were monitored solely during follow-up appointments [21].

### Regular foot care practice

In this study, foot care was significantly neglected, and few participants practiced daily washing and inspection. Most people overlook recommended practices such as washing with warm water and properly drying and caring for their feet. This problem has been frequently observed in Ethiopia, where foot care is one of the least recognized self-care practices [29,66–69]. A study in northeastern Ethiopia found that most patients neglected foot care due to a lack of proper guidance and knowledge [21]. A study in India revealed that foot inspection, specialized shoes, moisturizing, and nail care are frequently neglected [70].

Individuals with diabetes should wash their feet, dry them thoroughly, and pay close attention to their care [7]. The evidence suggests that effective foot care requires critical health literacy skills [71]. Enhancing knowledge, skills, and awareness of foot care is essential for improving practices [68].

### Barriers to diabetic self-management behaviors

The study found that insufficient knowledge is a major barrier, affecting patients’ ability to practice recommended self-care behaviors daily. This finding is consistently observed across global populations [23,72–76]. In Ethiopia, many people with diabetes lack fundamental knowledge of self-care practices, preventing their ability to manage their condition effectively [19,30].

This study identified lack of motivation, sadness, stress, and anger as key psychological barriers to self-care for individuals with diabetes. Many participants have lack of motivation to perform recommended self-management, which is consistent with previous research on challenges in adhering to physical activity, diet, and other health practices [23,72,74,77]. Studies in Ethiopia indicate that many individuals with diabetes demonstrate low personal motivation toward engaging in self-care practices [22,29–31].

In this study, financial constraints were found to directly hinder dietary modifications, physical activity, and glucose monitoring by limiting access to essential resources. Research has shown that low-income and affordability issues significantly influence diabetic self-care practices [54,76,78,79]. Ethiopian studies highlight that financial constraints significantly hinder diabetes self-care, particularly in terms of dietary adjustments, glucose monitoring, and exercise routines [20,30,63].

This current study found that self-care guidance and counseling were inadequate and inconsistent, resulting in unclear management plans. This is similar to findings in Nepal, where poor counseling also resulted in insufficient patient support and information [72]. In this study, a lack of family support emerged as a significant barrier, with patients making it challenging to maintain healthy meal plans because of the difficulty of preparing separate meals. Similar challenges have been reported in Ethiopia [29], Ghana [80], and India [81], where families often insist on their usual meals.

This study found that peer pressure and social events lead to poor self-care behavior, especially unhealthy eating. Similar studies in Ethiopia [19,29,31,49] and Pakistan [54] show that healthy food options are limited at social gatherings, which leads to poor dietary choices.

### Limitations

This study has some limitations that should be acknowledged. The participants were selected from a specific setting, which may not reflect the experiences of all individuals living with diabetes in other regions or contexts. Additionally, the potential for researcher bias exists, particularly during interpretation, despite efforts to ensure reflexivity and analytical rigor. Social desirability bias may also have influenced participants’ responses during interviews. Lastly, although data saturation was achieved, the findings are context-specific and should be interpreted accordingly.

### Implications for practice

Diabetic self-management continues to be a major challenge for many patients due to multiple complex and interrelated barriers. To address these issues, comprehensive community-based programs with culturally tailored behavioral change interventions that focus on diet, exercise, foot care, and glucose monitoring should be implemented. Healthcare providers play a key role in diabetes self-management by offering tailored education, counseling, and follow-up care. They help patients overcome psychological and social barriers, promote healthy behaviors, and involve family support. Providers also advocate for access to essential resources and collaborate with other professionals to ensure comprehensive, patient-centered care.

Mental health support and personalized guidance should be incorporated to address motivation and stress, and training for healthcare providers should be provided. Community and family support through partnerships and local initiatives should be strengthened, access to low-cost services should be improved, and policies that ensure equitable access to essential resources, including healthy food, medications, glucometers, educational materials, and exercise facilities, should be advocated.

## Conclusion

In the present study, diabetic self-management practices were inadequate, and the integration of recommended self-care into daily schedules was challenging. Although most patients adhere to their anti-diabetic medication, many face challenges with blood sugar monitoring, dietary practice, regular exercise, and foot care.

The study highlights that inadequate self-management is influenced by multiple barriers at the individual, interpersonal, and community levels. The most important barriers identified are lack of basic self-care knowledge, socioeconomic limitations, inadequate guidance and counseling, psychological factors such as low motivation and stress, limited social support, cultural factors, and limited access to essential resources.

## Supporting information

S1 File Inclusivity-in-global-research-questionnaire. (DOCX)

S2 File Transcribed data. (DOCX)

## Abbreviations

EC: Ethiopian calendar
T2DM: Type 2 diabetes mellitus
T1DM: Type 1 diabetes mellitus
