# Supplementary material for: Lived experiences of diabetes self-management in North Shoa, Ethiopia: A phenomenological inquiry
Source: PLoS One. 2026 Jun 30;21(6):e0316505. doi: 10.1371/journal.pone.0316505 (PMC13318053; doi:10.1371/journal.pone.0316505)
Supplement: S2 File — (DOCX) [file pone.0316505.s002.docx]

**Transcribed data for lived experience of self-management behavior North Shoa Zone**

**Respondent Code-1**

1. Socio demographic information

1. Sex- male,
2. Age 80,
3. residence urban ,
4. occupation merchant
5. type of Dm: Type-1

2. Transcribed data related to self-management behavior and barriers

- Life is hard now. What kind of self-care practices are there? I leave it day to day.
- I am not always followed the self-management practices recommended by a doctor.
- I don't do any physical exercise other than walking once a day.
- I know in some way the foods that are allowed for diabetics.
- There is no specific diet other than eating what I find.
- Lack of knowledge due to lack of understanding of the recommended practice
- I struggle to live on my own, and my family does not provide me with sufficient support.
- Providing professional advice and support is not enough and is mostly non-existent. In fact, it is not like they teach one day every six months.
- My blood sugar level will not increase if I do the recommended activities, but if I don't do it, it will increase.
- I regularly taking anti-diabetic medication. I don’t check my blood glucose levels at home. I don’t have the material and don’t know how to use one.

**Respondent Code-2**

1. Socio demographic information

1. Sex of respondent: male
2. Age: 32 years
3. Residence of the respondent: rural area
4. occupation: farmer
5. type of Dm: unknown

2. Transcribed data related to self-management behavior and barriers

- "In connection with my agricultural work, I do a lot of physical labor; I don't know the types of regular physical exercise that are allowed for diabetic patients.
- *I’m not familiar with different types of physical exercise beyond what my doctors have advised, which is walking. I lack detailed guidance on other exercises and how to perform them.*
- When I exercise and take a lot of medicine, my blood sugar level goes down, so I don't do regular physical exercise.
- When I experienced stress, I stopped to performing tasks/it becomes an obstacle to performing tasks.
- Lack of knowledge, Insufficient income, depressions are my obstacle /barrier to performing recommended actions/behaviors
- My family gives me enough support to follow recommended behaviors
- I take my medication properly. I don’t check my blood glucose levels at home. I don’t have the material and don’t know how to use one.
- Lack of suitable conditions for carrying out recommended activities in the area where I live
- There is no education supporter in our area to provide advice and support.
- I recommend to strengthen professional support by providing regular and adequate awareness-raising education and counseling services at least once per month (or preferably on a weekly basis).
- There is no community programs, and local support groups DM patient in my area.

**Respondent Code-3**

1. Socio demographic information

1. Sex of the respondent- male,
2. Age: 25 years
3. Residence of the respondent: rural area
4. occupation: private worker
5. type of Dm: Type-2

2. Transcribed data related to self-management behavior and barriers

- I follow my diet as prescribed by my doctor, I take my medication properly, and keep my feet clean.
- I know some foods that are allowed/recommended by physicians. I ate vegetables, fruits, and other recommended foods.
- I don't do any physical exercise, I perform *activities related to daily work*
- Even though I have some understanding, I do not have sufficient knowledge about different type recommended behaviors
- Lack of adequate provider advice and support, as well as lack of health education
- Lack of adequate provider advice and support, Lack of knowledge, Insufficient income are my obstacle /barrier to performing recommended actions/behaviors
- Lack of health education program also barrier to performing recommended actions/behaviors and information
- My family is concerned about my health and encourages me to maintain a healthy diet*.*
- Lack of sufficient psychological (motivation and readiness) to perform recommended behaviors

**Respondent Code-4**

1. Socio demographic information

1. Sex of the respondent- female,
2. Age: 54 years
3. Residence; urban ,
4. occupation: housewife
5. type of Dm: unknown

2. Transcribed data related to self-management behavior and barriers

- I still have difficulty following recommended self-care practices due to my social problems and the nature of the disease, which causes irritability
- I don't do any recommended physical exercise in my life, except for tasks around the house.
- I don't have enough knowledge about physical exercise. I don't know anything other than what doctors say to do walking.
- I eat as prescribed by my doctor. But occasionally I eat foods that are not allowed when I feel sick.
- I take care of my feet. But I don't know what kind of socks and shoes to wear.
- Unless I gets treatment, I do not give me adequate advice and education. In the past, health professionals used to teach us, but now I do not.
- Lack of sufficient motivation, life is boring these days, so I live like I'm at home.
- Lack of sufficient income, how can I followed. I don’t have the glucometer and don’t know how to use one.
- I have enough family support, my family understands me well, so I don't have any worries.
- The community should be made aware of these and support me and DM patients. Resources should be easily accessible for us

**Respondent Code-5**

1. Socio demographic information

1. Sex- male,
2. Age 22,
3. Residence: urban ,
4. occupation; private
5. type of Dm: Type-1

2. Transcribed data related to self-management behavior and barriers

- As people get older, they follow these practices to help them live longer, but since I am still young and don't think about dying, I don't follow the recommended self-care practices.
- *I still have difficulty following recommended self-care practices due to my social problems and the nature of the disease, which causes irritability.*
- *Even though I know the recommended foods, I eat forbidden foods, when having fun with friends, as my appetite takes over*
- *I don’t check my feet every day, but I do make an effort to keep them safe and healthy.*
- I told one history due to community pressure, he stopped taking his medication, spent a lot of time in bed, had high blood sugar levels, and suffered from foot ulcers and was chronically ill.
- I did not have good knowledge about recommended practices
- I did not performing activities recommended by the doctor due to lack of awareness
- I ate available foods, I am not being able to eat in a balanced manner.

**Respondent Code-6**

1. Socio demographic information

1. Sex of the respondent: male,
2. Age; 55 years
3. Residence: rural area
4. Occupation: government employee
5. type of Dm: Type-2

2. Transcribed data related to self-management behavior and barriers

- I still have difficulty following recommended self-care practices due to my social problems and the nature of the disease, which causes irritability.
- I wasn't doing the recommended activities when I first got sick with diabetes. But when my sugar levels became too high, I started to take the recommended action.
- *I don’t regularly inspect, wash, and dry my feet and haven’t considered these practices recommended for diabetic patients.*
- I stopped following self-care practices because of feelings of hopelessness and social issues. After my brother's death, I stopped taking my medication, and as a result, my blood sugar levels is not able to drop from 250 mg/dL.
- Even when I visit the hospital with a serious illness, I do not receive enough support and advice on my illness condition and the recommended activities to perform at home.
- *Even when I visit the hospital for a serious illness, I don't receive adequate support and advice on my condition and the recommended self-care activities to do at home.*
- The income problem has a major impact on the ability to eat affordable foods.
- Family support is sufficient and they take good care of me.
- I think if there is sufficient advice and support, this advice and support will save us.

**Respondent Code-7**

1. Socio demographic information

1. Sex- male,
2. Age 57,
3. Residence: rural ,
4. Occupation: farmer
5. type of Dm: Type-2

2. Transcribed data related to self-management behavior and barriers

- I have low financial income. How can I access the variety and types of food that are recommended for diabetic patients?
- *I wasn’t aware that diabetic patients are advised to self-monitor their blood glucose at home. But I take my medication properly,*
- I don't do any recommended activities, except for tasks related to agriculture.
- I do not have knowledge about different type recommended behaviors
- I ate available foods in my home. Preparing separate meals for a diabetic family member is challenging in my family.
- There is no education supporter in our area to provide advice and support. Our community is not supportive and no facilities
- Some community members believe that medication combined with daily exercise can lower blood glucose, which reduces my motivation to stay active.

**Respondent Code-8**

1. Socio demographic information

1. Sex of the respondent- male,
2. Age: 70 years
3. Residence; urban area
4. occupation: government employee
5. Type of Dm: Type-2

2. Transcribed data related to self-management behavior and barriers

- I don't do any work/practice because of diabetes, I just do walking exercises through practice and reading various written materials. I don't do anything every day.
- *I don't have any self-care practices related to diabetes management and care. Nothing I do every day.*
- I don't have any eating plan; I eat what I get without making specific choices to survive. Life is not what I think it is; there is no food that I can choose to eat.
- Since 2005 EC, I have not received adequate guidance and counseling on recommended self-care practices, except for the advice from physicians to avoid sweet foods and walk.
- I have sufficient family support. And they support take my medication properly,
- I need health education program that is appropriate to my level of awareness and education status
- We need experts/ educator to give us enough time to teach us.

**Respondent Code-9**

1. Socio demographic information

1. Sex of the respondents: female,
2. Age: 60 years
3. Residence: rural area
4. Occupation: housewife
5. type of Dm: unknown

2. Transcribed data related to self-management behavior and barriers

- My sugar level is rising because I eat whatever food I get. I eat milk, meat, butter, foods high in sugar, and various other foods.
- *At weddings, holidays, and religious ceremonies, suitable food for diabetics is often lacking, so I ate whatever is available.*
- I sometimes skip my injections during periods of fasting.
- When I feel hopeless and stressed, I eat whatever I find.
- I never eat food without salt
- I didn't do any recommended exercise, but I perform agriculture related activities related to daily work
- Family support is good and supports me. I take my medication properly
- There no health education program on recommended behaviors.
- There is no local support groups for diabetes management in my area.

**Respondent Code 10**

1. Socio demographic information

1. Sex of the respondent: female,
2. Age: 47 years
3. Residence: rural area
4. Occupation: housewife
5. Type of Dm: unknown

2. Transcribed data related to self-management behavior and barriers

- I didn't exercise regularly because I didn't have a stable life. In the future, I will try to follow the recommended self-care practices that are supposed to help me live a little longer.
- I did not pay attention to the disease and had a lack of attention to perform these recommended tasks.
- I don't know what types of food are allowed and forbidden. I just eat whatever I can find. There is no separate meals me
- When we come for treatment, there is no health professional who can give us any information about self-care practice. There is no health education whatsoever. It is similar to community level

**Respondent Code 11**

1. Socio demographic information

1. Sex- male,
2. Age 41,
3. residence urban ,
4. occupation: private
5. type of Dm: unknown

2. Transcribed data related to self-management behavior and barriers

- Since I was diagnosed with the disease, I avoid eating forbidden foods. I follow my diet as prescribed by my doctor.
- I know what foods are allowed and what foods are not allowed, even in moderation.
- I don't have any family support. I have had to handle everything on my own and manage myself.
- Even though the site where I receive my medication is injured, the doctor does not take the time to listen to my concerns. I doubt the effectiveness of the medication, as my blood sugar level has remained at 250 mg/dL for a long period.
- I do walking, but I don't do any other activities.
- I don’t check my blood glucose levels at home. I don’t have the material and don’t know how to use one.
- I take care of my feet, even if it's not always the case. I take my medication properly

**Respondent Code 12**

1. Socio demographic information

1. Sex- female,
2. Age of the respondent: 25 years
3. Residence: urban area
4. Occupation: government employee
5. type of Dm: unknown

2. Transcribed data related to self-management behavior and barriers

- I walk, but I do not do any other exercise. I didn't know the recommended exercises for a person with diabetes.
- I don't know the dietary guidelines. I know some recommended and forbidden foods, although to a limited extent. I eat leafy greens, fruits, vegetables, and other recommended foods.
- I take care to keep my feet clean and protect them from injury. I also take my medication properly
- I didn't follow self-care practice due lack of time and lack of attention
- Health care workers give not attention on advice and support. The professional's focus is on the blood sugar number, there is no support.
- Health care provider did not understand the patient's pain and need
- My motivation is expressed as my sense of pain.
- I have good Family support, they support and encourage me. But the community member also support DM pt. There are no community programs, and local support in my area.

**Respondent Code 13**

1. Socio demographic information

1. Sex of the respondent- male,
2. Age: 52 years
3. Residence: urban area
4. Occupation: Private worker
5. Type of Dm: Type-2

2. Transcribed data related to self-management behavior and barriers

- I don't know the recommended exercises for diabetics. But I walk as much as I can. I exercise 2 or 3 days a week.
- *I didn't participate in regular moderate-intensity exercise because I didn't know what exercises to do.*
- *Some community members believe that starting diabetes medication implies there is no cure and that diabetes will be permanent.*
- I also know a little about what foods are allowed and what foods are not allowed.
- I have some what little foot care practice
- There is a problem related to knowledge about recommended behavior
- There is limited support and advice services by health care provider.

**Respondent Code 14**

1. Socio demographic information

1. Sex of the respondents: male,
2. Age of the respondents: 54 yaers
3. Residence: rural area
4. Occupation: government employee
5. type of Dm: Type-2

2. Transcribed data related to self-management behavior and barriers

- The physician told me to exercise, but I don't know what exercises they meant. They didn't give me clear instructions.
- *I’m not familiar with different types of physical exercise beyond what my doctors have advised, which is walking* and also *I perform agriculture related activities*
- *I understand the importance of a healthy diet, but it's challenging to prepare separate diabetic-friendly meals from what my family eats.*
- Even if I don't eat sweet and fatty foods and foods high in sugar, I don't follow the recommended diet because I cannot find the recommended foods.
- *At weddings, holidays, and religious ceremonies, suitable food for diabetics is often lacking, so I end up eating whatever is available.*
- There are not enough facilities in our area to accommodate those who are. I don’t have the glucometer.
- There is also a problem of attitude. If our attitude is good, there will be motivation to do anything.
- There is a problem with access to permitted foods. And also there is no local support groups from the community in my area.
- I live in a remote area where diabetes management resources are not easily accessible
- I have good family support and support me to take my medication properly,

**Respondent Code 15**

1. Socio demographic information

1. Sex- male,
2. Age of the respondent: 28 years
3. Residence: Rural,
4. Occupation: agriculture /framer
5. type of Dm: Unknown

2. Transcribed data related to self-management behavior and barriers

- I have activities related to my daily work, but I do not engage in the regular physical exercise recommended for diabetic patients.
- *I don’t regularly check my feet and don’t know about the recommended foot care practices for diabetic patients.*
- *I feared that starting medication meant accepting diabetes as a lifelong condition. I stopped my medication, which led to complications and leg ulcers*
- *I don't follow recommended practices due to negligence, impatience, and anger, which leads to frequent illness and high blood sugar levels.*
- Due to community pressure, I discontinued my medication, which led to complications and leg ulcers.
- I know to some extent what foods are allowed and what foods are not.
- Income instability mainly affect my practice related to recommended behavior. And also resources are not easily accessible
- I have adequate family support. When I feel my blood sugar level dropping, my family quickly gives me foods that increase my blood sugar level.

**Respondent Code 16**

1. Socio demographic information

1. Sex- female,
2. Age 23
3. residence rural ,
4. occupation: daily worker
5. type of Dm: Type-1

2. Transcribed data related to self-management behavior and barriers

- I did not participate in any regular physical exercise because I didn't know what exercises to do.
- *"I didn't participate in regular moderate-intensity exercise because I didn't know what exercises to do.*
- I stopped my medication (injection) when I ran out of medicine. I cannot move to the hospital due to instability in my district.
- I don't use the recommended self-management behaviors because I have no patience and I get angry. As a result, my sugar level increases and I get sick frequently.
- I know, to a certain extent, what foods are allowed and what foods are not.
- I didn't do foot care practice.
- There is no professional support, they focus on treatment
- I did not receive health education on recommended behavior
- I don't have family support, my family doesn't support me.
- There is no community programs, and local support groups in my area. I live in a remote area where diabetes management resources are not easily accessible

**Respondent Code 17**

1. Socio demographic information

1. Sex- female
2. Age 60,
3. residence urban ,
4. occupation; housewife
5. type of Dm: unknown

2. Transcribed data related to self-management behavior and barriers

- I walk some days, but I don't do any physical exercise except walking.
- I walk, I don't eat foods that increase sugar levels. I don't do any physical exercise other than walking, and I don't know of any.
- I know what foods are allowed and what foods are not allowed, although in a certain way. I eat vegetables and fruits and other recommended foods.
- I take care of foot care.
- I have a limited knowledge and have not received any health education to provide any advice or support.
- I did not getting enough information. I come to the medical center, I get treated, l leave, but health care provider don't give us any information.
- Limited Income affect my practice.
- I have a low socioeconomic status and limited income, making it difficult to afford a healthy diet and a glucometer due to the high costs. How can I adhere to self-care practices under these conditions?

**Respondent Code 18**

1. Socio demographic information

1. Sex- male,
2. Age: 50 years
3. Residence: urban ,
4. Occupation: government employee
5. type of Dm: Type-1

2. Transcribed data related to self-management behavior and barriers

- I exercise three days a week.
- I don't eat forbidden foods that raise my sugar levels. I eat vegetables, fruits, and other recommended foods.
- *Even if healthcare providers are busy, they should offer education on nutrition, exercise, and other self-care practices at least once a month.*
- I get information in some way, even if it is not directly from an health care provider
- I do have foot care practice. I take my medication but, I don’t check my blood glucose levels because I don’t have the material and don’t know how to use one.
- My family is concerned about my health and encourages me to maintain a healthy diet. I have good family support and encourage me
- Health care provider should teach us at least once a year about nutrition and physical fitness.

**Respondent Code 19**

1. Socio demographic information

1. Sex- female,
2. Age 56,
3. residence urban ,
4. occupation: housewife
5. type of Dm: unknown

2. Transcribed data related to self-management behavior and barriers

- I don’t have adequate information on the types and content of foods recommended for diabetic patients. I just eat what I find.
- *I understand the importance of a healthy diet, but I eat whatever is prepared at home. Making separate meals for myself and my family is too challenging.*
- *Some community members believe that starting diabetes medication implies there is no cure and that diabetes will be permanent.*
- I don't regularly check my feet. I don’t have any information on the regular check and safety of feet recommended to diabetic patients
- health professional must give repeated health education and community programs, and local support groups in my area also need

**Respondent Code 20**

1. Socio demographic information

1. Sex- female,
2. Age of the respondents: 45 years
3. Residence: urban area
4. occupation; government employee
5. type of Dm: Type-2

2. Transcribed data related to self-management behavior and barriers

- *I’m not familiar with different types of physical exercise beyond what my doctors have advised, which is walking. I lack detailed guidance on other exercises and how to perform them.*
- I don't have regularly check my feet. I don’t have any information on the regular check and safety of feet recommended to diabetic patients
- *It’s important to check my blood glucose when I feel unwell, but I didn’t measure it because I don’t have a glucometer and don’t know how to use one.*
- I take my medication properly
- I did not have information about what foods are allowed and what foods are not allowed.
- I did not follow recommended due to lack of knowledge, income status and availability
- I think community support is important but there are no community programs, and local support groups for diabetes management in my area.
