# Supplementary material for: Lived experiences of diabetes self-management in North Shoa, Ethiopia: A phenomenological inquiry
Source: PLoS One. 2026 Jun 30;21(6):e0316505. doi: 10.1371/journal.pone.0316505 (PMC13318053; doi:10.1371/journal.pone.0316505)
Supplement: Attachment — Submitted filename: Authors response to Reviewer.pdf [file pone.0316505.s004.pdf]

Dear Editorial Board Member**!**

PLOS ONE

Response to Editor Comments

I am happy to resubmit for revised version of "**Exploring the Lived Experiences of Diabetes Self-Management Behaviors: A Phenomenological Study in North Shoa Zone, Ethiopia**". We have addressed your concerns point by point and highlighted changes to the manuscript.

Thank you for your consideration of this manuscript!

Journal Requirements:

When submitting your revision, we need you to address these additional requirements.

1. Please ensure that your manuscript meets PLOS ONE's style requirements, including those for file naming. The PLOS ONE style templates can be found at https://journals.plos.org/plosone/s/file?id=wjVg/PLOSOne_formatting_sample_main_body.pdf andhttps://journals.plos.org/plosone/s/file?id=ba62/PLOSOne_formatting_sample_title_authors_affiliations.pf

2. Please include a complete copy of PLOS' questionnaire on inclusivity in global research in your revised manuscript. Our policy for research in this area aims to improve transparency in the reporting of research performed outside of researchers' own country or community. The policy applies to researchers who have travelled to a different country to conduct research, research with Indigenous populations or their lands, and research on cultural artefacts. The questionnaire can also be requested at the journal's discretion for any other submissions, even if these conditions are not met. Please find more information on the policy and a link to download a blank copy of the questionnaire here: https://journals.plos.org/plosone/s/best-practices-in-research-reporting. Please upload a completed version of your questionnaire as Supporting Information when you resubmit your manuscript.

Response: We did based on the comments

3. Your ethics statement should only appear in the Methods section of your manuscript. If your ethics statement is written in any section besides the Methods, please move it to the Methods section and delete it from any other section. Please ensure that your ethics statement is included in your manuscript, as the ethics statement entered into the online submission form will not be published alongside your manuscript.

Response: ethic statement is written under declaration section based on the format

4. Please include captions for your Supporting Information files at the end of your manuscript, and update any in-text citations to match accordingly. Please see our Supporting Information guidelines for more information: http://journals.plos.org/plosone/s/supporting-information.

5. Please review your reference list to ensure that it is complete and correct. If you have cited papers that have been retracted, please include the rationale for doing so in the manuscript text, or remove these references and replace them with relevant current references. Any changes to the reference list should be mentioned in the rebuttal letter that accompanies your revised manuscript. If you need to cite a retracted article, indicate the article's retracted status in the References list and also include a citation and full reference for the retraction notice.

Additional Editor Comments:

Dear Authors,
Thank you for submitting your manuscript titled "Exploring the Lived Experiences of Diabetes Self-Management Behaviors: A Phenomenological Study in North Shoa Zone, Ethiopia" to PLOS ONE. We appreciate your contribution to understanding diabetes self-care practices through qualitative inquiry. After careful review, we have compiled constructive feedback to help guide revisions.
Title
• The title is clear but slightly long.
• Conciseness improves readability and impact.
• Consider: 'Lived Experiences of Diabetes Self-Management in North Shoa, Ethiopia: A Phenomenological Inquiry'.

Response: we did based on the comments
Abstract
• The abstract is generally well-structured but contains some repetition.
• Avoiding redundancy can make the abstract more impactful and easier to follow.
• Refrain from repeating 'barriers' and briefly indicate key themes.

Response: we did based on the comments
Introduction
• The introduction is well-referenced but somewhat repetitive.
• Reducing redundancy enhances clarity and makes the research gap more prominent.
• Consolidate statements about global and national diabetes burden and end with a clear research aim.

Response: Thank you for your valuable feedback. We appreciate your insight on the introduction being well-referenced but somewhat repetitive. I will work on reducing redundancy to improve clarity and better highlight the research gap. I'll also consolidate the statements on the global and national diabetes burden and conclude with a clear research aim as you suggested.

Methodology

• The methodology is well described but could benefit from additional detail.

• Clarifying rationale and procedures ensures reproducibility and transparency.

Response: The methodology section clearly documents the methods, tools, data sources, and analysis steps to ensure reproducibility. At the same time, it outlines every aspect of the research process—data collection, analysis, and interpretation—while disclosing all relevant details, including limitations, assumptions, and potential conflicts of interest, to ensure transparency and build trust in the study.

• Provide more justification for the sample size and describe interview questions briefly.

Response: thank very much, in qualitative research, the aim is depth rather than breadth. The sample size was determined based on the principle of data saturation—the point at which no new themes or insights emerge from additional interviews. A manageable number of participants (e.g., 10–20) was selected to allow for rich, in-depth exploration of individual experiences while ensuring the feasibility of data collection and analysis. This sample size is adequate for capturing diverse perspectives related to diabetes self-management. Regarding, the interview questions were designed to explore participants' lived experiences with diabetes self-management. They covered key domains including diet and nutrition, physical activity, medication adherence, blood glucose monitoring, and foot care. Questions also probed into challenges faced, coping strategies, and perceived support from family, community, and healthcare providers. The open-ended format encouraged detailed personal narratives and reflections (page 6)

Results

• Results are thematically rich but can be streamlined.

• Too many long quotes can dilute key messages.

• Use representative, concise quotes and connect them to sociodemographic characteristics.

Response: thank very much these constrictive comments, we did accordingly.

Discussion

• The discussion aligns well with existing literature but could be more analytical.

• Analytical depth adds value by interpreting findings in broader contexts.

Response: thank very much these constrictive comments, we did accordingly.

• Incorporate frameworks like the Health Belief Model to interpret barriers.

Response: Although interpretation at this stage is challenging, the Health Belief Model (HBM) was used to help understand how individual beliefs—such as perceived severity, susceptibility, benefits, and barriers—influence self-care behaviors and decision-making in diabetes management.

Conclusion

• The conclusion is clear but reiterates prior content.

• A concise summary with a strong take-home message is preferable.

• Focus on the implications of multi-level interventions in a single paragraph.

Response: thank very much these constrictive comments, we did accordingly.

Reviewers' comments:

Comments to the Author

1. Is the manuscript technically sound, and do the data support the conclusions?

The manuscript must describe a technically sound piece of scientific research with data that supports the conclusions. Experiments must have been conducted rigorously, with appropriate controls, replication, and sample sizes. The conclusions must be drawn appropriately based on the data presented.

Reviewer #1: Yes

Reviewer #2: Yes

---

2. Has the statistical analysis been performed appropriately and rigorously?

Reviewer #1: Yes

Reviewer #2: Yes

---

3. Have the authors made all data underlying the findings in their manuscript fully available?

The PLOS Data policy requires authors to make all data underlying the findings described in their manuscript fully available without restriction, with rare exception (please refer to the Data Availability Statement in the manuscript PDF file). The data should be provided as part of the manuscript or its supporting information, or deposited to a public repository. For example, in addition to summary statistics, the data points behind means, medians and variance measures should be available. If there are restrictions on publicly sharing data—e.g. participant privacy or use of data from a third party—those must be specified.

Reviewer #1: Yes

Reviewer #2: No

---

4. Is the manuscript presented in an intelligible fashion and written in standard English?

PLOS ONE does not copyedit accepted manuscripts, so the language in submitted articles must be clear, correct, and unambiguous. Any typographical or grammatical errors should be corrected at revision, so please note any specific errors here.

Reviewer #1: Yes

Reviewer #2: Yes

---

5. Review Comments to the Author

Please use the space provided to explain your answers to the questions above. You may also include additional comments for the author, including concerns about dual publication, research ethics, or publication ethics. (Please upload your review as an attachment if it exceeds 20,000 characters)

1. Reviewer #1: Abstract Clarity: The abstract effectively summarizes the study but could benefit from a clearer statement of the research objectives and key findings. Consider explicitly stating how the study's findings contribute to the existing body of knowledge.

Response: thank you very much, we did based on the comments

2. Grammar and Syntax: There are several grammatical errors throughout the manuscript. For example, in the Abstract, the phrase "AMR tends to be one of the greatest threats..." should be revised to "AMR is widely recognized as one of the greatest threats...". A thorough proofreading is recommended.

Response: thank you very much, we did based on the comments

3. Sampling Method Justification: The use of maximum variation sampling is appropriate, but the rationale for selecting 20 diabetic patients and five healthcare informants should be better justified. Explain how this sample size ensures data saturation.

Response: thank you very much, data saturation was reached when no new themes or insights emerged during consecutive interviews, particularly after the 19th patient and the 4th healthcare informant. At that point, information became repetitive, indicating that additional interviews were unlikely to yield new findings. Therefore, the sample size was deemed sufficient to achieve a comprehensive understanding of the research questions within the study context.

4. Methodological Detail: More detail is needed regarding the data analysis process with ATLAS.ti. Specifically, explain how the thematic framework was applied and provide examples of coding steps.

Response: Thank you. The data analysis process is detailed on page 7. The recorded interviews were transcribed into English, and appropriate codes were generated. From these, subthemes and main themes related to diabetes self-care and its barriers were developed. The initial analysis identified five key subthemes and 18 corresponding codes.

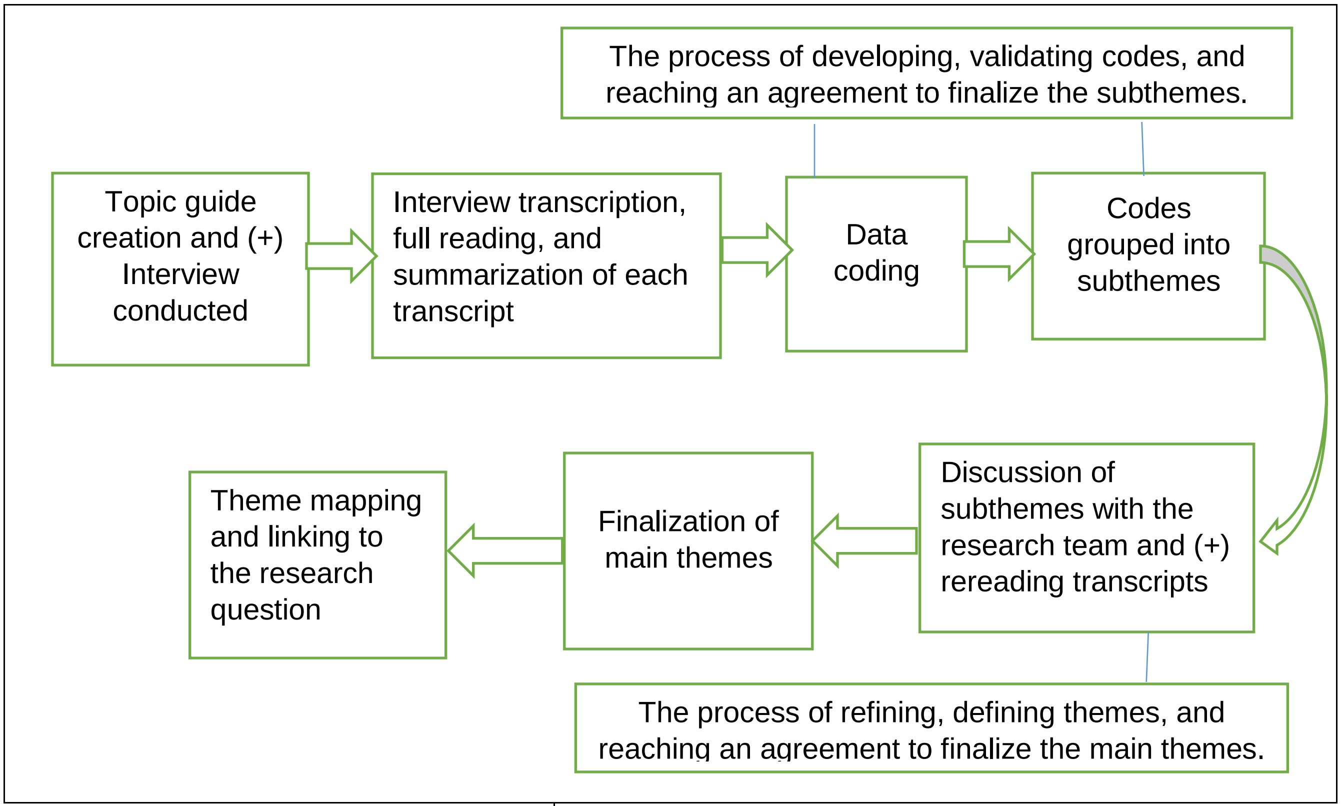

Main theme	***Subtheme/categories***	**Selected coded statement**
Adherence to self-management behaviors	Regular physical exercise	○ Exercised 2-3 days a week<br>○ Walking is the only way to exercise.<br>○ Doing activities related to daily work<br>○ Do not do any physical exercise in their life including walking.<br>○ No consideration of intensity-moderate activities
	Dietary practice	○ Didn't have a healthy eating plan in place<br>○ Despite lacking a weekly plan, they mainly eat vegetables, fruits and other recommended foods.<br>○ Did not consume enough fruits and vegetables, evenly space carbohydrates, and include foods with low glycemic index.<br>○ Eating what they got without making specific choices
	Regularly taking anti-diabetic medication.	○ Regularly taking anti-diabetic medication.<br>○ Skipping medication or injections during fasting

		○ Stopping medication due to instability in their area
	Self-blood glucose monitoring	○ Did not regularly check their blood glucose levels<br>○ Lacks a glucometer and is unfamiliar with its use
	Foot care practice	○ Wash and inspect their feet every day.<br>○ Did not regularly check their feet every day<br>○ Inconsistent health checks and washing

5. Participant Demographics: In the Results section, the sociodemographic characteristics are presented, but their relevance to the study findings is not fully explored. Discuss how factors such as age, sex, and socioeconomic status influenced self-management behaviors.

Response: thank you very much. The finding is narrated and quoted by considering the sociodemographic variables.

6. Presentation of Results: While participant quotes enrich the narrative, some sections are overloaded with quotes without sufficient analysis. Consider balancing direct quotes with analytical insights to strengthen the discussion.

Response: thank you very much. We accepted the comments and minimize overloaded quotes.

7.Themes and Subthemes: The main themes and subthemes are well-identified, but the connection between subthemes and overarching themes could be clearer. A visual representation (e.g., a thematic map) might help readers understand these relationships.

Response: thank you very much. A summary table was developed to present findings across self-care behavior domains, while a thematic map was used to illustrate key barriers to diabetes self-care. Both were appropriately referenced within the text.

8.Ethical Considerations: The ethics statement is appropriate, but further clarification on how participant confidentiality was maintained during data collection and analysis would strengthen this section.

Response: thank you very much. We did accordingly the comments

9.Discussion Depth: The discussion could be more critically engaged with existing literature.

While some comparisons are made, a deeper analysis of how these findings align or contrast with previous studies would enhance the discussion.

Response: thank you very much. We did accordingly the comments. The findings were interpreted in relation to existing literature during our discussion.

10. Implications for Practice: The manuscript discusses the need for community-based interventions but could provide more concrete recommendations for how healthcare providers can support diabetes self-management in similar settings.

Response: thank you very much. We did accordingly the comments. We included recommendations on how healthcare providers can support diabetes self-management in similar settings.

11. Conclusion Strength: The conclusion reiterates the findings but lacks a strong closing statement on the broader implications for healthcare policy and practice. Consider emphasizing the need for targeted interventions and policy support.

Response: thank you very much. We did accordingly the comments

12. Typographical Errors: There are minor typographical errors, such as "meaasures" instead of "measures" and "tranferable" instead of "transferable." Careful proofreading is necessary.

Response: thank you very much. We did accordingly the comments

13. Reference Accuracy: Ensure all in-text citations correspond to the reference list. Some claims in the Results and Discussion sections lack proper citation.

Response: thank you very much. We did accordingly the comments

14. Data Saturation: While the manuscript mentions data saturation, it would be helpful to specify how this was determined during data collection.

Response: thank very much, Data saturation was considered reached when no significant information emerged from successive interviews. During data collection, the research team continuously reviewed and analyzed the data after each interview. Once responses became repetitive and additional interviews yielded no new insights relevant to the research questions, saturation was judged to have been achieved. This typically occurred after 20 interviews.

15. Limitations: The manuscript would benefit from a more explicit limitations section, discussing potential biases, sample size constraints, and generalizability of the findings.

Response: thank you very much. We did accordingly the comments (page 21)

16. Figure and Table Presentation: Ensure all figures and tables are properly labeled and referenced in the text. A visual summary of key findings could enhance reader comprehension.

Response: thank you very much. We checked accordingly the comments

Reviewer #2: Study Overview, Abstract and Introduction

This study makes a meaningful contribution to the existing body of research on diabetes mellitus as a global health condition, specifically addressing challenges and barriers to diabetes management with the stated purpose to "promote effective behavioral change and achieve personal health goals, thereby improving diabetes self-management." The abstract clearly articulates the study's objectives and provides appropriate context for the research focus on the Northern Shoa zone of Ethiopia.

Recommendations:
In the introduction background section, the statement "In Ethiopia, poor diabetes self-management remains an important public health issue, despite government efforts" requires specification of what categories or specific government efforts are referenced. Providing examples would help contextualize the government's role, either in this section or in the discussion when addressing practical implications.

Response: thank you very much. We did accordingly. The Government of Ethiopia has implemented various initiatives, including providing treatment through primary healthcare facilities and developing strategic plans for the prevention and control of major non-communicable diseases, as referenced in the main manuscript.

Throughout multiple sections in the abstract and paper, the phrase "Diabetic self-management practices were insufficient" needs clarification regarding what specific outcomes this insufficiency pertains to. Based on the manuscript, I believe this refers to adherence to treatment and effective self-care management, but this needs to be explicitly clarified.

Response: thank you very much. Diabetes self-management includes regular blood glucose monitoring, adherence to dietary recommendations, physical activity, and proper foot care. Based on the findings, although most patients consistently take their anti-diabetic medications, many

struggle with maintaining other essential self-care practices, particularly glucose monitoring, healthy eating, exercise, and foot care.

1. Original Research

While this study addresses an important topic, the originality lies primarily in the specific geographic focus on Northern Shoa rather than the broader research question. Extensive literature exists on barriers to diabetes care globally, which the authors acknowledge in the discussion but should also briefly mention in the introduction for accuracy and proper contextualization.

Response: thank you very much. We did based on the concerns

2. Novelty of Results

The novelty of this research is appropriately situated in its representation of the North Shoa zone specifically, rather than the overall topic area. This geographic specificity should be more clearly articulated to accurately represent the study's unique contribution.

3. Technical Standards and Methodology

The methodology demonstrates several strengths: the rationale for using phenomenological study design is well-justified, the principle of data saturation appropriately guided the number of interviews, and maximum variation sampling was appropriate for capturing a wide range of self-care experiences and challenges. The acknowledgment of researcher reflexivity and mitigation processes is commendable.

Response: thank you very much. We did based on the concerns

Recommendations:

1. Clarify the rationale for the sample size of 20 patients—why was a larger sample size not pursued?

Response: thank very much, in qualitative research, the aim is depth rather than breadth. The sample size was determined based on the principle of data saturation—the point at which no new themes or insights emerge from additional interviews. A manageable number of participants (e.g., 10–20) was selected to allow for rich, in-depth exploration of individual experiences while ensuring the feasibility of data collection and analysis. This sample size is adequate for capturing diverse perspectives related to diabetes self-management. Regarding, the interview questions were designed to explore participants' lived experiences with diabetes self-management. They covered

key domains including diet and nutrition, physical activity, medication adherence, blood glucose monitoring, and foot care. Questions also probed into challenges faced, coping strategies, and perceived support from family, community, and healthcare providers. The open-ended format encouraged detailed personal narratives and reflections (page 6)

2. Consider acknowledging the role of researcher gender in reflexivity, as this may also require recognition.

Response: thank you very much. We did based on the concerns

Specify how consent was obtained (written or verbal).

Response: thank you very much. Written informed consent was obtained from all participants. To ensure confidentiality, personal identifiers were removed, and anonymous codes were used during both data collection and analysis. Participants' rights to withdraw or decline to answer any questions were fully respected, and strict data protection protocols were maintained throughout the study (Page 22).

Clarify who constituted the peer review cohort referenced in "The findings were also peer reviewed before finalization."

Response: thank you very much, authors are peer reviewed the findings.

4. Conclusions and Data Support; The study's conclusions are generally well-supported by the data collected. The connections made between sub-theme findings and comparable research from other countries strengthen the analysis.

Response: thank you very much, we considered the concerns at discussion part.
Critiques and Recommendations:
Quantify references to "only a few" and "most" in each sub-theme for better understanding, especially given the small cohort size.

Response: thank you very much, we considered most if the respondents responds more than ¾ participants in the same concerns.

Consider incorporating approaches from other countries that could be referenced in the "Implications for Practice" section, given the comparable sub-themes identified internationally. Given the mention of government role in public health efforts in the introduction, explore potential government roles alongside the proposed community-based program roles.

Response: thank you very much, we did accordingly the concern

5. Overall Presentation

The manuscript is generally well-written and intelligible, adhering to standard English conventions.

Recommendation:

Table 3 requires formatting consistency—some statements include periods while others do not.

Response: thank you very much, we did accordingly the concern

6. Ethical Standards and Integrity

The study demonstrates strong adherence to ethical standards and research integrity through its incorporation of trained data collectors, study supervision, expert opinion consultation, translative and validation efforts, and appropriate patient consenting procedures.

Recommendation:

For enhanced transparency, provide further clarification of the process referenced in "The interview guide was developed through comprehensive literature and consultations with experts in the field."

Response: thank you very much, we did accordingly the concern

7. Reporting Guidelines and Data Availability

The study appropriately makes mapping data available, meeting standards for data accessibility. However, there is no data repository for the interview guide. Please clarify if one should not exist.

Response: thank you very much, we attached the interview guide

Final Assessment

This research contributes valuable geographic-specific insights to the existing literature on diabetes self-management barriers and challenges. While the broader topic has been studied, the focus on Northern Shoa, Ethiopia, provides important contextual understanding that can inform targeted interventions. The study merits publication with minor revisions addressing the methodological clarifications and presentation issues outlined above.

6. PLOS authors have the option to publish the peer review history of their article (what does this mean?). If published, this will include your full peer review and any attached files.

If you choose “no”, your identity will remain anonymous but your review may still be made public.

Do you want your identity to be public for this peer review? For information about this choice, including consent withdrawal, please see our Privacy Policy.

Reviewer #1: **Yes:** ADEWUNMI AKINGBOLA

Reviewer #2: No

[NOTE: If reviewer comments were submitted as an attachment file, they will be attached to this email and accessible via the submission site. Please log into your account, locate the manuscript record, and check for the action link "View Attachments". If this link does not appear, there are no attachment files.]

While revising your submission, please upload your figure files to the Preflight Analysis and Conversion Engine (PACE) digital diagnostic tool, https://pacev2.apexcovantage.com/. PACE helps ensure that figures meet PLOS requirements. To use PACE, you must first register as a user. Registration is free. Then, login and navigate to the UPLOAD tab, where you will find detailed instructions on how to use the tool. If you encounter any issues or have any questions when using PACE, please email PLOS at figures@plos.org. Please note that Supporting Information files do not need this step.

In compliance with data protection regulations, you may request that we remove your personal registration details at any time. (Remove my information/details). Please contact the publication office if you have any questions.
