# Supplementary material for: Lived experiences of diabetes self-management in North Shoa, Ethiopia: A phenomenological inquiry
Source: PLoS One. 2026 Jun 30;21(6):e0316505. doi: 10.1371/journal.pone.0316505 (PMC13318053; doi:10.1371/journal.pone.0316505)
Supplement: Attachment — Submitted filename: Response to data publication consent and restrictions..pdf [file pone.0316505.s006.pdf]

Dear Editorial Board Member**!**

PLOS ONE

Response to Editor Comments

I am happy to resubmit for revised version of ***"Lived Experiences of Diabetes Self-Management in North Shoa, Ethiopia: A Phenomenological Inquiry."*** We have addressed your concerns point by point.

Current Comments

We've checked your submission and before we can proceed, we need you to address the following issues:

1. Thank you for providing this information about the data associated with your study and your Data Availability Statement. We have one clarification before we proceed with your submission.

PLOS One requires that data request contacts be a non-author. Could you please provide us with a non-author contact for your Data Availability Statement?

Alternatively, with the data that you have provided in Supporting Information and within the manuscript itself, you can choose to have the Data Availability Statement be "All relevant data are within the manuscript and its Supporting Information files." Please let me know what you would like to proceed with for your Data Availability Statement?

Response

Thank you for your request regarding our manuscript entitled "Lived Experiences of Diabetes Self-Management in North Shoa, Ethiopia: A Phenomenological Inquiry. In response, we have provided a non-author contact for the Data Availability Statement. This individual is not a co-author of the manuscript.

Non-author Data Request Contact:

- Name: Asmamaw Abera Kebedde
- Title: Lecturer, PhD candidate at Addis Ababa University
- Institution: Asrat Woldeyes Health Science Campus, Debre Berhan University
- Email:asmenst13@gmail.com
- Phone: +251911002936
