# Supplementary material for: Lived experiences of diabetes self-management in North Shoa, Ethiopia: A phenomenological inquiry
Source: PLoS One. 2026 Jun 30;21(6):e0316505. doi: 10.1371/journal.pone.0316505 (PMC13318053; doi:10.1371/journal.pone.0316505)
Supplement: Peer review history

# Peer review: Decision Letter 0

**DOI:** 10.1371/journal.pone.0316505.r001

**Authors:** Ammal Metwally

9 Jun 2025

PONE-D-24-55247

Exploring the Lived Experiences of Diabetes Self-Management Behaviors: A Phenomenological Study in North Shoa Zone, Ethiopia

PLOS ONE

Dear Dr. Eshete,

Thank you for submitting your manuscript to PLOS ONE. After careful consideration, we feel that it has merit but does not fully meet PLOS ONE’s publication criteria as it currently stands. Therefore, we invite you to submit a revised version of the manuscript that addresses the points raised during the review process.

Please submit your revised manuscript by Jul 24 2025 11:59PM. If you will need more time than this to complete your revisions, please reply to this message or contact the journal office at plosone@plos.org. When you're ready to submit your revision, log on to https://www.editorialmanager.com/pone/ and select the 'Submissions Needing Revision' folder to locate your manuscript file.

Please include the following items when submitting your revised manuscript:

- A rebuttal letter that responds to each point raised by the academic editor and reviewer(s). You should upload this letter as a separate file labeled 'Response to Reviewers'.
- A marked-up copy of your manuscript that highlights changes made to the original version. You should upload this as a separate file labeled 'Revised Manuscript with Track Changes'.
- An unmarked version of your revised paper without tracked changes. You should upload this as a separate file labeled 'Manuscript'.

-->If you would like to make changes to your financial disclosure, please include your updated statement in your cover letter. Guidelines for resubmitting your figure files are available below the reviewer comments at the end of this letter.

If applicable, we recommend that you deposit your laboratory protocols in protocols.io to enhance the reproducibility of your results. Protocols.io assigns your protocol its own identifier (DOI) so that it can be cited independently in the future. For instructions see: https://journals.plos.org/plosone/s/submission-guidelines#loc-laboratory-protocols. Additionally, PLOS ONE offers an option for publishing peer-reviewed Lab Protocol articles, which describe protocols hosted on protocols.io. Read more information on sharing protocols at https://plos.org/protocols?utm_medium=editorial-email&utm_source=authorletters&utm_campaign=protocols.

We look forward to receiving your revised manuscript.

Kind regards,

Ammal Mokhtar Metwally, Ph.D (MD)

Academic Editor

PLOS ONE

Journal Requirements:

When submitting your revision, we need you to address these additional requirements.

1. Please ensure that your manuscript meets PLOS ONE's style requirements, including those for file naming. The PLOS ONE style templates can be found at

https://journals.plos.org/plosone/s/file?id=wjVg/PLOSOne_formatting_sample_main_body.pdf and

https://journals.plos.org/plosone/s/file?id=ba62/PLOSOne_formatting_sample_title_authors_affiliations.pdf

2. Please include a complete copy of PLOS’ questionnaire on inclusivity in global research in your revised manuscript. Our policy for research in this area aims to improve transparency in the reporting of research performed outside of researchers’ own country or community. The policy applies to researchers who have travelled to a different country to conduct research, research with Indigenous populations or their lands, and research on cultural artefacts. The questionnaire can also be requested at the journal’s discretion for any other submissions, even if these conditions are not met.  Please find more information on the policy and a link to download a blank copy of the questionnaire here: https://journals.plos.org/plosone/s/best-practices-in-research-reporting. Please upload a completed version of your questionnaire as Supporting Information when you resubmit your manuscript.

3. Your ethics statement should only appear in the Methods section of your manuscript. If your ethics statement is written in any section besides the Methods, please move it to the Methods section and delete it from any other section. Please ensure that your ethics statement is included in your manuscript, as the ethics statement entered into the online submission form will not be published alongside your manuscript.

4. Please include captions for your Supporting Information files at the end of your manuscript, and update any in-text citations to match accordingly. Please see our Supporting Information guidelines for more information: http://journals.plos.org/plosone/s/supporting-information.

5. Please review your reference list to ensure that it is complete and correct. If you have cited papers that have been retracted, please include the rationale for doing so in the manuscript text, or remove these references and replace them with relevant current references. Any changes to the reference list should be mentioned in the rebuttal letter that accompanies your revised manuscript. If you need to cite a retracted article, indicate the article’s retracted status in the References list and also include a citation and full reference for the retraction notice.

Additional Editor Comments:

Dear Authors,

Thank you for submitting your manuscript titled “Exploring the Lived Experiences of Diabetes Self-Management Behaviors: A Phenomenological Study in North Shoa Zone, Ethiopia” to PLOS ONE. We appreciate your contribution to understanding diabetes self-care practices through qualitative inquiry. After careful review, we have compiled constructive feedback to help guide revisions.

Title

• The title is clear but slightly long.

• Conciseness improves readability and impact.

• Consider: 'Lived Experiences of Diabetes Self-Management in North Shoa, Ethiopia: A Phenomenological Inquiry'.

Abstract

• The abstract is generally well-structured but contains some repetition.

• Avoiding redundancy can make the abstract more impactful and easier to follow.

• Refrain from repeating 'barriers' and briefly indicate key themes.

Introduction

• The introduction is well-referenced but somewhat repetitive.

• Reducing redundancy enhances clarity and makes the research gap more prominent.

• Consolidate statements about global and national diabetes burden and end with a clear research aim.

Methodology

• The methodology is well described but could benefit from additional detail.

• Clarifying rationale and procedures ensures reproducibility and transparency.

• Provide more justification for the sample size and describe interview questions briefly.

Results

• Results are thematically rich but can be streamlined.

• Too many long quotes can dilute key messages.

• Use representative, concise quotes and connect them to sociodemographic characteristics.

Discussion

• The discussion aligns well with existing literature but could be more analytical.

• Analytical depth adds value by interpreting findings in broader contexts.

• Incorporate frameworks like the Health Belief Model to interpret barriers.

Conclusion

• The conclusion is clear but reiterates prior content.

• A concise summary with a strong take-home message is preferable.

• Focus on the implications of multi-level interventions in a single paragraph.

We look forward to receiving a revised version of this manuscript that incorporates the suggested improvements.

[Note: HTML markup is below. Please do not edit.]

Reviewers' comments:

Reviewer's Responses to Questions

**Comments to the Author**

1. Is the manuscript technically sound, and do the data support the conclusions?

The manuscript must describe a technically sound piece of scientific research with data that supports the conclusions. Experiments must have been conducted rigorously, with appropriate controls, replication, and sample sizes. The conclusions must be drawn appropriately based on the data presented.

Reviewer #1: Yes

Reviewer #2: Yes

**********

2. Has the statistical analysis been performed appropriately and rigorously?

Reviewer #1: Yes

Reviewer #2: Yes

**********

3. Have the authors made all data underlying the findings in their manuscript fully available?

The PLOS Data policy requires authors to make all data underlying the findings described in their manuscript fully available without restriction, with rare exception (please refer to the Data Availability Statement in the manuscript PDF file). The data should be provided as part of the manuscript or its supporting information, or deposited to a public repository. For example, in addition to summary statistics, the data points behind means, medians and variance measures should be available. If there are restrictions on publicly sharing data—e.g. participant privacy or use of data from a third party—those must be specified.

Reviewer #1: Yes

Reviewer #2: No

**********

4. Is the manuscript presented in an intelligible fashion and written in standard English?

PLOS ONE does not copyedit accepted manuscripts, so the language in submitted articles must be clear, correct, and unambiguous. Any typographical or grammatical errors should be corrected at revision, so please note any specific errors here.

Reviewer #1: Yes

Reviewer #2: Yes

**********

5. Review Comments to the Author

Please use the space provided to explain your answers to the questions above. You may also include additional comments for the author, including concerns about dual publication, research ethics, or publication ethics. (Please upload your review as an attachment if it exceeds 20,000 characters)

Reviewer #1: Abstract Clarity: The abstract effectively summarizes the study but could benefit from a clearer statement of the research objectives and key findings. Consider explicitly stating how the study's findings contribute to the existing body of knowledge.

Grammar and Syntax: There are several grammatical errors throughout the manuscript. For example, in the Abstract, the phrase "AMR tends to be one of the greatest threats..." should be revised to "AMR is widely recognized as one of the greatest threats...". A thorough proofreading is recommended.

Sampling Method Justification: The use of maximum variation sampling is appropriate, but the rationale for selecting 20 diabetic patients and five healthcare informants should be better justified. Explain how this sample size ensures data saturation.

Methodological Detail: More detail is needed regarding the data analysis process with ATLAS.ti. Specifically, explain how the thematic framework was applied and provide examples of coding steps.

Participant Demographics: In the Results section, the sociodemographic characteristics are presented, but their relevance to the study findings is not fully explored. Discuss how factors such as age, sex, and socioeconomic status influenced self-management behaviors.

Presentation of Results: While participant quotes enrich the narrative, some sections are overloaded with quotes without sufficient analysis. Consider balancing direct quotes with analytical insights to strengthen the discussion.

Themes and Subthemes: The main themes and subthemes are well-identified, but the connection between subthemes and overarching themes could be clearer. A visual representation (e.g., a thematic map) might help readers understand these relationships.

Ethical Considerations: The ethics statement is appropriate, but further clarification on how participant confidentiality was maintained during data collection and analysis would strengthen this section.

Discussion Depth: The discussion could be more critically engaged with existing literature. While some comparisons are made, a deeper analysis of how these findings align or contrast with previous studies would enhance the discussion.

Implications for Practice: The manuscript discusses the need for community-based interventions but could provide more concrete recommendations for how healthcare providers can support diabetes self-management in similar settings.

Conclusion Strength: The conclusion reiterates the findings but lacks a strong closing statement on the broader implications for healthcare policy and practice. Consider emphasizing the need for targeted interventions and policy support.

Typographical Errors: There are minor typographical errors, such as "meaasures" instead of "measures" and "tranferable" instead of "transferable." Careful proofreading is necessary.

Reference Accuracy: Ensure all in-text citations correspond to the reference list. Some claims in the Results and Discussion sections lack proper citation.

Data Saturation: While the manuscript mentions data saturation, it would be helpful to specify how this was determined during data collection.

Limitations: The manuscript would benefit from a more explicit limitations section, discussing potential biases, sample size constraints, and generalizability of the findings.

Figure and Table Presentation: Ensure all figures and tables are properly labeled and referenced in the text. A visual summary of key findings could enhance reader comprehension.

Reviewer #2: Study Overview, Abstract and Introduction

This study makes a meaningful contribution to the existing body of research on diabetes mellitus as a global health condition, specifically addressing challenges and barriers to diabetes management with the stated purpose to "promote effective behavioral change and achieve personal health goals, thereby improving diabetes self-management." The abstract clearly articulates the study's objectives and provides appropriate context for the research focus on the Northern Shoa zone of Ethiopia.

Recommendations:

In the introduction background section, the statement "In Ethiopia, poor diabetes self-management remains an important public health issue, despite government efforts" requires specification of what categories or specific government efforts are referenced. Providing examples would help contextualize the government's role, either in this section or in the discussion when addressing practical implications.

Throughout multiple sections in the abstract and paper, the phrase "Diabetic self-management practices were insufficient" needs clarification regarding what specific outcomes this insufficiency pertains to. Based on the manuscript, I believe this refers to adherence to treatment and effective self-care management, but this needs to be explicitly clarified.

1. Original Research

While this study addresses an important topic, the originality lies primarily in the specific geographic focus on Northern Shoa rather than the broader research question. Extensive literature exists on barriers to diabetes care globally, which the authors acknowledge in the discussion but should also briefly mention in the introduction for accuracy and proper contextualization.

2. Novelty of Results

The novelty of this research is appropriately situated in its representation of the North Shoa zone specifically, rather than the overall topic area. This geographic specificity should be more clearly articulated to accurately represent the study's unique contribution.

3. Technical Standards and Methodology

The methodology demonstrates several strengths: the rationale for using phenomenological study design is well-justified, the principle of data saturation appropriately guided the number of interviews, and maximum variation sampling was appropriate for capturing a wide range of self-care experiences and challenges. The acknowledgment of researcher reflexivity and mitigation processes is commendable.

Recommendations:

Clarify the rationale for the sample size of 20 patients—why was a larger sample size not pursued?

Consider acknowledging the role of researcher gender in reflexivity, as this may also require recognition.

Specify how consent was obtained (written or verbal).

Clarify who constituted the peer review cohort referenced in "The findings were also peer reviewed before finalization."

4. Conclusions and Data Support

The study's conclusions are generally well-supported by the data collected. The connections made between sub-theme findings and comparable research from other countries strengthen the analysis.

Critiques and Recommendations:

Quantify references to "only a few" and "most" in each sub-theme for better understanding, especially given the small cohort size.

Consider incorporating approaches from other countries that could be referenced in the "Implications for Practice" section, given the comparable sub-themes identified internationally.

Given the mention of government role in public health efforts in the introduction, explore potential government roles alongside the proposed community-based program roles.

5. Overall Presentation

The manuscript is generally well-written and intelligible, adhering to standard English conventions.

Recommendation:

Table 3 requires formatting consistency—some statements include periods while others do not.

6. Ethical Standards and Integrity

The study demonstrates strong adherence to ethical standards and research integrity through its incorporation of trained data collectors, study supervision, expert opinion consultation, translative and validation efforts, and appropriate patient consenting procedures.

Recommendation:

For enhanced transparency, provide further clarification of the process referenced in "The interview guide was developed through comprehensive literature and consultations with experts in the field."

7. Reporting Guidelines and Data Availability

The study appropriately makes mapping data available, meeting standards for data accessibility. However, there is no data repository for the interview guide. Please clarify if one should not exist.

Final Assessment

This research contributes valuable geographic-specific insights to the existing literature on diabetes self-management barriers and challenges. While the broader topic has been studied, the focus on Northern Shoa, Ethiopia, provides important contextual understanding that can inform targeted interventions. The study merits publication with minor revisions addressing the methodological clarifications and presentation issues outlined above.

**********

6. PLOS authors have the option to publish the peer review history of their article (what does this mean?). If published, this will include your full peer review and any attached files.

If you choose “no”, your identity will remain anonymous but your review may still be made public.

**Do you want your identity to be public for this peer review?**  For information about this choice, including consent withdrawal, please see our Privacy Policy.

Reviewer #1: **Yes:**  ADEWUNMI AKINGBOLA

Reviewer #2: No

**********

[NOTE: If reviewer comments were submitted as an attachment file, they will be attached to this email and accessible via the submission site. Please log into your account, locate the manuscript record, and check for the action link "View Attachments". If this link does not appear, there are no attachment files.]

While revising your submission, please upload your figure files to the Preflight Analysis and Conversion Engine (PACE) digital diagnostic tool, https://pacev2.apexcovantage.com/. PACE helps ensure that figures meet PLOS requirements. To use PACE, you must first register as a user. Registration is free. Then, login and navigate to the UPLOAD tab, where you will find detailed instructions on how to use the tool. If you encounter any issues or have any questions when using PACE, please email PLOS at figures@plos.org. Please note that Supporting Information files do not need this step.

# Author comment: Author response to Decision Letter 1

**DOI:** 10.1371/journal.pone.0316505.r002

1 Aug 2025

Dear Editorial Board Member!

PLOS ONE

Response to Editor Comments

I am happy to resubmit for revised version of “Exploring the Lived Experiences of Diabetes Self-Management Behaviors: A Phenomenological Study in North Shoa Zone, Ethiopia”. We have addressed your concerns point by point and highlighted changes to the manuscript.

Thank you for your consideration of this manuscript!

Journal Requirements:

When submitting your revision, we need you to address these additional requirements.

1. Please ensure that your manuscript meets PLOS ONE's style requirements, including those for file naming. The PLOS ONE style templates can be found at https://journals.plos.org/plosone/s/file?id=wjVg/PLOSOne_formatting_sample_main_body.pdf andhttps://journals.plos.org/plosone/s/file?id=ba62/PLOSOne_formatting_sample_title_authors_affiliations.pf

2. Please include a complete copy of PLOS’ questionnaire on inclusivity in global research in your revised manuscript. Our policy for research in this area aims to improve transparency in the reporting of research performed outside of researchers’ own country or community. The policy applies to researchers who have travelled to a different country to conduct research, research with Indigenous populations or their lands, and research on cultural artefacts. The questionnaire can also be requested at the journal’s discretion for any other submissions, even if these conditions are not met. Please find more information on the policy and a link to download a blank copy of the questionnaire here: https://journals.plos.org/plosone/s/best-practices-in-research-reporting. Please upload a completed version of your questionnaire as Supporting Information when you resubmit your manuscript.

Response: We did based on the comments

3. Your ethics statement should only appear in the Methods section of your manuscript. If your ethics statement is written in any section besides the Methods, please move it to the Methods section and delete it from any other section. Please ensure that your ethics statement is included in your manuscript, as the ethics statement entered into the online submission form will not be published alongside your manuscript.

Response: ethic statement is written under declaration section based on the format

4. Please include captions for your Supporting Information files at the end of your manuscript, and update any in-text citations to match accordingly. Please see our Supporting Information guidelines for more information: http://journals.plos.org/plosone/s/supporting-information.

5. Please review your reference list to ensure that it is complete and correct. If you have cited papers that have been retracted, please include the rationale for doing so in the manuscript text, or remove these references and replace them with relevant current references. Any changes to the reference list should be mentioned in the rebuttal letter that accompanies your revised manuscript. If you need to cite a retracted article, indicate the article’s retracted status in the References list and also include a citation and full reference for the retraction notice.

Additional Editor Comments:

Dear Authors,

Thank you for submitting your manuscript titled “Exploring the Lived Experiences of Diabetes Self-Management Behaviors: A Phenomenological Study in North Shoa Zone, Ethiopia” to PLOS ONE. We appreciate your contribution to understanding diabetes self-care practices through qualitative inquiry. After careful review, we have compiled constructive feedback to help guide revisions.

Title

• The title is clear but slightly long.

• Conciseness improves readability and impact.

• Consider: 'Lived Experiences of Diabetes Self-Management in North Shoa, Ethiopia: A Phenomenological Inquiry'.

Response: we did based on the comments

Abstract

• The abstract is generally well-structured but contains some repetition.

• Avoiding redundancy can make the abstract more impactful and easier to follow.

• Refrain from repeating 'barriers' and briefly indicate key themes.

Response: we did based on the comments

Introduction

• The introduction is well-referenced but somewhat repetitive.

• Reducing redundancy enhances clarity and makes the research gap more prominent.

• Consolidate statements about global and national diabetes burden and end with a clear research aim.

Response: Thank you for your valuable feedback. We appreciate your insight on the introduction being well-referenced but somewhat repetitive. I will work on reducing redundancy to improve clarity and better highlight the research gap. I’ll also consolidate the statements on the global and national diabetes burden and conclude with a clear research aim as you suggested.

Methodology

• The methodology is well described but could benefit from additional detail.

• Clarifying rationale and procedures ensures reproducibility and transparency.

Response: The methodology section clearly documents the methods, tools, data sources, and analysis steps to ensure reproducibility. At the same time, it outlines every aspect of the research process—data collection, analysis, and interpretation—while disclosing all relevant details, including limitations, assumptions, and potential conflicts of interest, to ensure transparency and build trust in the study.

• Provide more justification for the sample size and describe interview questions briefly.

Response: thank very much, in qualitative research, the aim is depth rather than breadth. The sample size was determined based on the principle of data saturation—the point at which no new themes or insights emerge from additional interviews. A manageable number of participants (e.g., 10–20) was selected to allow for rich, in-depth exploration of individual experiences while ensuring the feasibility of data collection and analysis. This sample size is adequate for capturing diverse perspectives related to diabetes self-management. Regarding, the interview questions were designed to explore participants’ lived experiences with diabetes self-management. They covered key domains including diet and nutrition, physical activity, medication adherence, blood glucose monitoring, and foot care. Questions also probed into challenges faced, coping strategies, and perceived support from family, community, and healthcare providers. The open-ended format encouraged detailed personal narratives and reflections (page 6)

Results

• Results are thematically rich but can be streamlined.

• Too many long quotes can dilute key messages.

• Use representative, concise quotes and connect them to sociodemographic characteristics.

Response: thank very much these constrictive comments, we did accordingly.

Discussion

• The discussion aligns well with existing literature but could be more analytical.

• Analytical depth adds value by interpreting findings in broader contexts.

Response: thank very much these constrictive comments, we did accordingly.

• Incorporate frameworks like the Health Belief Model to interpret barriers.

Response: Although interpretation at this stage is challenging, the Health Belief Model (HBM) was used to help understand how individual beliefs—such as perceived severity, susceptibility, benefits, and barriers—influence self-care behaviors and decision-making in diabetes management.

Conclusion

• The conclusion is clear but reiterates prior content.

• A concise summary with a strong take-home message is preferable.

• Focus on the implications of multi-level interventions in a single paragraph.

Response: thank very much these constrictive comments, we did accordingly.

Reviewers' comments:

Comments to the Author

1. Is the manuscript technically sound, and do the data support the conclusions?

The manuscript must describe a technically sound piece of scientific research with data that supports the conclusions. Experiments must have been conducted rigorously, with appropriate controls, replication, and sample sizes. The conclusions must be drawn appropriately based on the data presented.

Reviewer #1: Yes

Reviewer #2: Yes

________________________________________

2. Has the statistical analysis been performed appropriately and rigorously?

Reviewer #1: Yes

Reviewer #2: Yes

________________________________________

3. Have the authors made all data underlying the findings in their manuscript fully available?

The PLOS Data policy requires authors to make all data underlying the findings described in their manuscript fully available without restriction, with rare exception (please refer to the Data Availability Statement in the manuscript PDF file). The data should be provided as part of the manuscript or its supporting information, or deposited to a public repository. For example, in addition to summary statistics, the data points behind means, medians and variance measures should be available. If there are restrictions on publicly sharing data—e.g. participant privacy or use of data from a third party—those must be specified.

Reviewer #1: Yes

Reviewer #2: No

________________________________________

4. Is the manuscript presented in an intelligible fashion and written in standard English?

PLOS ONE does not copyedit accepted manuscripts, so the language in submitted articles must be clear, correct, and unambiguous. Any typographical or grammatical errors should be corrected at revision, so please note any specific errors here.

Reviewer #1: Yes

Reviewer #2: Yes

________________________________________

5. Review Comments to the Author

Please use the space provided to explain your answers to the questions above. You may also include additional comments for the author, including concerns about dual publication, research ethics, or publication ethics. (Please upload your review as an attachment if it exceeds 20,000 characters)

1. Reviewer #1: Abstract Clarity: The abstract effectively summarizes the study but could benefit from a clearer statement of the research objectives and key findings. Consider explicitly stating how the study's findings contribute to the existing body of knowledge.

Response: thank you very much, we did based on the comments

2. Grammar and Syntax: There are several grammatical errors throughout the manuscript. For example, in the Abstract, the phrase "AMR tends to be one of the greatest threats..." should be revised to "AMR is widely recognized as one of the greatest threats...". A thorough proofreading is recommended.

Response: thank you very much, we did based on the comments

3. Sampling Method Justification: The use of maximum variation sampling is appropriate, but the rationale for selecting 20 diabetic patients and five healthcare informants should be better justified. Explain how this sample size ensures data saturation.

Response: thank you very much, data saturation was reached when no new themes or insights emerged during consecutive interviews, particularly after the 19th patient and the 4th healthcare informant. At that point, information became repetitive, indicating that additional interviews were unlikely to yield new findings. Therefore, the sample size was deemed sufficient to achieve a comprehensive understanding of the research questions within the study context.

4. Methodological Detail: More detail is needed regarding the data analysis process with ATLAS.ti. Specifically, explain how the thematic framework was applied and provide examples of coding steps.

Response: Thank you. The data analysis process is detailed on page 7. The recorded interviews were transcribed into English, and appropriate codes were generated. From these, subthemes and main themes related to diabetes self-care and its barriers were developed. The initial analysis identified five key subthemes and 18 corresponding codes.

Main theme Subtheme/categories Selected coded statement

Adherence to self-management behaviors Regular physical exercise o Exercised 2-3 days a week

o Walking is the only way to exercise.

o Doing activities related to daily work

o Do not do any physical exercise in their life including walking.

o No consideration of intensity-moderate activities

Dietary practice o Didn’t have a healthy eating plan in place

o Despite lacking a weekly plan, they mainly eat vegetables, fruits and other recommended foods.

o Did not consume enough fruits and vegetables, evenly space carbohydrates, and include foods with low glycemic index.

o Eating what they got without making specific choices

Regularly taking anti-diabetic medication. o Regularly taking anti-diabetic medication.

o Skipping medication or injections during fasting

o Stopping medication due to instability in their area

Self-blood glucose monitoring o Did not regularly check their blood glucose levels

o Lacks a glucometer and is unfamiliar with its use

Foot care practice o Wash and inspect their feet every day.

o Did not regularly check their feet every day

o Inconsistent health checks and washing

5. Participant Demographics: In the Results section, the sociodemographic characteristics are presented, but their relevance to the study findings is not fully explored. Discuss how factors such as age, sex, and socioeconomic status influenced self-management behaviors.

Response: thank you very much. The finding is narrated and quoted by considering the sociodemographic variables.

6. Presentation of Results: While participant quotes enrich the narrative, some sections are overloaded with quotes without sufficient analysis. Consider balancing direct quotes with analytical insights to strengthen the discussion.

Response: thank you very much. We accepted the comments and minimize overloaded quotes.

7.Themes and Subthemes: The main themes and subthemes are well-identified, but the connection between subthemes and overarching themes could be clearer. A visual representation (e.g., a thematic map) might help readers understand these relationships.

Response: thank you very much. A summary table was developed to present findings across self-care behavior domains, while a thematic map was used to illustrate key barriers to diabetes self-care. Both were appropriately referenced within the text.

8.Ethical Considerations: The ethics statement is appropriate, but further clarification on how participant confidentiality was maintained during data collection and analysis would strengthen this section.

Response: thank you very much. We did accordingly the comments

9.Discussion Depth: The discussion could be more critically engaged with existing literature. While some comparisons are made, a deeper analysis of how these findings align or contrast with previous studies would enhance the discussion.

Response: thank you very much. We did accordingly the comments. The findings were interpreted in relation to existing literature during our discussion.

10. Implications for Practice: The manuscript discusses the need for community-based interventions but could provide more concrete recommendations for how healthcare providers can support diabetes self-management in similar settings.

Response: thank you very much. We did accordingly the comments. We included recommendations on how healthcare providers can support diabetes self-management in similar settings.

11. Conclusion Strength: The conclusion reiterates the findings but lacks a strong closing statement on the broader implications for healthcare policy and practice. Consider emphasizing the need for targeted interventions and policy support.

Response: thank you very much. We did accordingly the comments

12. Typographical Errors: There are minor typographical errors, such as "meaasures" instead of "measures" and "tranferable" instead of "transferable." Careful proofreading is necessary.

Response: thank you very much. We did accordingly the comments

13. Reference Accuracy: Ensure all in-text citations correspond to the reference list. Some claims in the Results and Discussion sections lack proper citation.

Response: thank you very much. We did accordingly the comments

14. Data Saturation: While the manuscript mentions data saturation, it would be helpful to specify how this was determined during data collection.

Response: thank very much, Data saturation was considered reached when no significant information

Attachment Submitted filename: Authors response to Reviewer.pdf

# Peer review: Decision Letter 1

**DOI:** 10.1371/journal.pone.0316505.r003

**Authors:** Ammal Metwally, Ammal Metwally

19 Oct 2025

PONE-D-24-55247R1

Lived Experiences of Diabetes Self-Management in North Shoa, Ethiopia: A Phenomenological Inquiry

PLOS ONE

Dear Dr. Eshete,

Thank you for submitting your manuscript to PLOS ONE. After careful consideration, we feel that it has merit but does not fully meet PLOS ONE’s publication criteria as it currently stands. Therefore, we invite you to submit a revised version of the manuscript that addresses the points raised during the review process.

Please submit your revised manuscript by Dec 03 2025 11:59PM. If you will need more time than this to complete your revisions, please reply to this message or contact the journal office at plosone@plos.org. When you're ready to submit your revision, log on to https://www.editorialmanager.com/pone/ and select the 'Submissions Needing Revision' folder to locate your manuscript file.

Please include the following items when submitting your revised manuscript:

- A rebuttal letter that responds to each point raised by the academic editor and reviewer(s). You should upload this letter as a separate file labeled 'Response to Reviewers'.
- A marked-up copy of your manuscript that highlights changes made to the original version. You should upload this as a separate file labeled 'Revised Manuscript with Track Changes'.
- An unmarked version of your revised paper without tracked changes. You should upload this as a separate file labeled 'Manuscript'.

-->If you would like to make changes to your financial disclosure, please include your updated statement in your cover letter. Guidelines for resubmitting your figure files are available below the reviewer comments at the end of this letter.

If applicable, we recommend that you deposit your laboratory protocols in protocols.io to enhance the reproducibility of your results. Protocols.io assigns your protocol its own identifier (DOI) so that it can be cited independently in the future. For instructions see: https://journals.plos.org/plosone/s/submission-guidelines#loc-laboratory-protocols. Additionally, PLOS ONE offers an option for publishing peer-reviewed Lab Protocol articles, which describe protocols hosted on protocols.io. Read more information on sharing protocols at https://plos.org/protocols?utm_medium=editorial-email&utm_source=authorletters&utm_campaign=protocols.

We look forward to receiving your revised manuscript.

Kind regards,

Ammal Mokhtar Metwally, Ph.D (MD)

Academic Editor

PLOS ONE

Journal Requirements:

1. If the reviewer comments include a recommendation to cite specific previously published works, please review and evaluate these publications to determine whether they are relevant and should be cited. There is no requirement to cite these works unless the editor has indicated otherwise.

2. Please review your reference list to ensure that it is complete and correct. If you have cited papers that have been retracted, please include the rationale for doing so in the manuscript text, or remove these references and replace them with relevant current references. Any changes to the reference list should be mentioned in the rebuttal letter that accompanies your revised manuscript. If you need to cite a retracted article, indicate the article’s retracted status in the References list and also include a citation and full reference for the retraction notice.

Additional Editor Comments:

Some responses are repetitive (we did accordingly”) without quoting or summarizing the revised text itself, which makes it difficult to verify the exact depth of change.

While the authors state compliance, a few responses could benefit from brief illustrative phrases (e.g., quoting one revised sentence or summarizing the new addition).

[Note: HTML markup is below. Please do not edit.]

Reviewers' comments:

Reviewer's Responses to Questions

**Comments to the Author**

1. If the authors have adequately addressed your comments raised in a previous round of review and you feel that this manuscript is now acceptable for publication, you may indicate that here to bypass the “Comments to the Author” section, enter your conflict of interest statement in the “Confidential to Editor” section, and submit your "Accept" recommendation.

Reviewer #2: (No Response)

**********

2. Is the manuscript technically sound, and do the data support the conclusions?

The manuscript must describe a technically sound piece of scientific research with data that supports the conclusions. Experiments must have been conducted rigorously, with appropriate controls, replication, and sample sizes. The conclusions must be drawn appropriately based on the data presented.

Reviewer #2: Yes

**********

3. Has the statistical analysis been performed appropriately and rigorously?

Reviewer #2: Yes

**********

4. Have the authors made all data underlying the findings in their manuscript fully available?

The PLOS Data policy requires authors to make all data underlying the findings described in their manuscript fully available without restriction, with rare exception (please refer to the Data Availability Statement in the manuscript PDF file). The data should be provided as part of the manuscript or its supporting information, or deposited to a public repository. For example, in addition to summary statistics, the data points behind means, medians and variance measures should be available. If there are restrictions on publicly sharing data—e.g. participant privacy or use of data from a third party—those must be specified.

Reviewer #2: Yes

**********

5. Is the manuscript presented in an intelligible fashion and written in standard English?

PLOS ONE does not copyedit accepted manuscripts, so the language in submitted articles must be clear, correct, and unambiguous. Any typographical or grammatical errors should be corrected at revision, so please note any specific errors here.

Reviewer #2: Yes

**********

6. Review Comments to the Author

Please use the space provided to explain your answers to the questions above. You may also include additional comments for the author, including concerns about dual publication, research ethics, or publication ethics. (Please upload your review as an attachment if it exceeds 20,000 characters)

Reviewer #2: Formatting critique:

In discussion has two commas - please correct:

Regularly taking anti-diabetic medication. In this study, most participants regularly took their anti-diabetic medication, which is consistent with findings from Ethiopia, where patients generally demonstrate better adherence [31]. The study revealed that some participants had difficulties fasting and experienced medication shortages due to instability. Similar findings reported in prior studies, , which

**********

7. PLOS authors have the option to publish the peer review history of their article (what does this mean?). If published, this will include your full peer review and any attached files.

If you choose “no”, your identity will remain anonymous but your review may still be made public.

**Do you want your identity to be public for this peer review?**  For information about this choice, including consent withdrawal, please see our Privacy Policy.

Reviewer #2: No

**********

[NOTE: If reviewer comments were submitted as an attachment file, they will be attached to this email and accessible via the submission site. Please log into your account, locate the manuscript record, and check for the action link "View Attachments". If this link does not appear, there are no attachment files.]

While revising your submission, please upload your figure files to the Preflight Analysis and Conversion Engine (PACE) digital diagnostic tool, https://pacev2.apexcovantage.com/. PACE helps ensure that figures meet PLOS requirements. To use PACE, you must first register as a user. Registration is free. Then, login and navigate to the UPLOAD tab, where you will find detailed instructions on how to use the tool. If you encounter any issues or have any questions when using PACE, please email PLOS at figures@plos.org. Please note that Supporting Information files do not need this step.

# Author comment: Author response to Decision Letter 2

**DOI:** 10.1371/journal.pone.0316505.r004

3 Dec 2025

Dear Editorial Board Member!

PLOS ONE

Response to Editor Comments

I am happy to resubmit for revised version of “Lived Experiences of Diabetes Self-Management in North Shoa, Ethiopia: A Phenomenological Inquiry.” We have addressed your concerns point by point. I am writing to inform you that my previous supervisor, Dr. Hendry Van Der Heever, has sadly passed away. He has been replaced by Prof. David D. Mphuthi by the University of South Africa. Therefore, I kindly request that Prof. David D. Mphuthi be included in the author list as my current supervisor.

Thank you for your understanding and kind consideration.

Response to Reviewers and Academic Editor

PONE-D-24-55247R1

Lived Experiences of Diabetes Self-Management in North Shoa, Ethiopia: A Phenomenological Inquiry

PLOS ONE

Dear Dr. Eshete,

Thank you for submitting your manuscript to PLOS ONE. After careful consideration, we feel that it has merit but does not fully meet PLOS ONE’s publication criteria as it currently stands. Therefore, we invite you to submit a revised version of the manuscript that addresses the points raised during the review process.

Please submit your revised manuscript by Dec 03 2025 11:59PM. If you will need more time than this to complete your revisions, please reply to this message or contact the journal office at plosone@plos.org. When you're ready to submit your revision, log on to https://www.editorialmanager.com/pone/ and select the 'Submissions Needing Revision' folder to locate your manuscript file.

Please include the following items when submitting your revised manuscript:

• A rebuttal letter that responds to each point raised by the academic editor and reviewer(s). You should upload this letter as a separate file labeled 'Response to Reviewers'.

• A marked-up copy of your manuscript that highlights changes made to the original version. You should upload this as a separate file labeled 'Revised Manuscript with Track Changes'.

• An unmarked version of your revised paper without tracked changes. You should upload this as a separate file labeled 'Manuscript'.

If you would like to make changes to your financial disclosure, please include your updated statement in your cover letter. Guidelines for resubmitting your figure files are available below the reviewer comments at the end of this letter.

If applicable, we recommend that you deposit your laboratory protocols in protocols.io to enhance the reproducibility of your results. Protocols.io assigns your protocol its own identifier (DOI) so that it can be cited independently in the future. For instructions see: https://journals.plos.org/plosone/s/submission-guidelines#loc-laboratory-protocols. Additionally, PLOS ONE offers an option for publishing peer-reviewed Lab Protocol articles, which describe protocols hosted on protocols.io. Read more information on sharing protocols at https://plos.org/protocols?utm_medium=editorial-email&utm_source=authorletters&utm_campaign=protocols.

We look forward to receiving your revised manuscript.

Kind regards,

Ammal Mokhtar Metwally, Ph.D (MD)

Academic Editor

PLOS ONE

Journal Requirements:

1. If the reviewer comments include a recommendation to cite specific previously published works, please review and evaluate these publications to determine whether they are relevant and should be cited. There is no requirement to cite these works unless the editor has indicated otherwise

Response: Thank you for the guidance. I reviewed the publications suggested by the reviewer and evaluated their relevance to my manuscript. Where they offered meaningful contributions to the context and strengthened the discussion, I have incorporated them accordingly previously

2. Please review your reference list to ensure that it is complete and correct. If you have cited papers that have been retracted, please include the rationale for doing so in the manuscript text, or remove these references and replace them with relevant current references. Any changes to the reference list should be mentioned in the rebuttal letter that accompanies your revised manuscript. If you need to cite a retracted article, indicate the article’s retracted status in the References list and also include a citation and full reference for the retraction notice.

Response: Thank you very much for the comments. We have thoroughly reviewed the reference list to ensure completeness and accuracy. Only a few references remained unchanged. Regarding the retracted articles, we included them based on the relevance of their findings to our results, as assessed through their abstracts. If the use of such references is not acceptable, we will remove them from the reference list.

Additional Editor Comments:

Some responses are repetitive (we did accordingly”) without quoting or summarizing the revised text itself, which makes it difficult to verify the exact depth of change. While the authors state compliance, a few responses could benefit from brief illustrative phrases (e.g., quoting one revised sentence or summarizing the new addition).

Response. Thank you for this valuable feedback. Due to the extensive revisions made throughout the manuscript, we were unable to quote every modification in the rebuttal letter. However, we fully recognize the importance of providing illustrative phrases or brief summaries to enhance clarity. We will certainly incorporate this approach in future submissions to ensure our revisions are easier to verify.

[Note: HTML markup is below. Please do not edit.]

Reviewers' comments:

Reviewer's Responses to Questions

Comments to the Author

1. If the authors have adequately addressed your comments raised in a previous round of review and you feel that this manuscript is now acceptable for publication, you may indicate that here to bypass the “Comments to the Author” section, enter your conflict of interest statement in the “Confidential to Editor” section, and submit your "Accept" recommendation.

Reviewer #2: (No Response)

________________________________________

2. Is the manuscript technically sound, and do the data support the conclusions?

The manuscript must describe a technically sound piece of scientific research with data that supports the conclusions. Experiments must have been conducted rigorously, with appropriate controls, replication, and sample sizes. The conclusions must be drawn appropriately based on the data presented.

Reviewer #2: Yes

________________________________________

3. Has the statistical analysis been performed appropriately and rigorously?

Reviewer #2: Yes

________________________________________

4. Have the authors made all data underlying the findings in their manuscript fully available?

The PLOS Data policy requires authors to make all data underlying the findings described in their manuscript fully available without restriction, with rare exception (please refer to the Data Availability Statement in the manuscript PDF file). The data should be provided as part of the manuscript or its supporting information, or deposited to a public repository. For example, in addition to summary statistics, the data points behind means, medians and variance measures should be available. If there are restrictions on publicly sharing data—e.g. participant privacy or use of data from a third party—those must be specified.

Reviewer #2: Yes

________________________________________

5. Is the manuscript presented in an intelligible fashion and written in standard English?

PLOS ONE does not copyedit accepted manuscripts, so the language in submitted articles must be clear, correct, and unambiguous. Any typographical or grammatical errors should be corrected at revision, so please note any specific errors here.

Reviewer #2: Yes

________________________________________

6. Review Comments to the Author

Please use the space provided to explain your answers to the questions above. You may also include additional comments for the author, including concerns about dual publication, research ethics, or publication ethics. (Please upload your review as an attachment if it exceeds 20,000 characters)

Reviewer #2: Formatting critique:

In discussion has two commas - please correct:

Regularly taking anti-diabetic medication. In this study, most participants regularly took their anti-diabetic medication, which is consistent with findings from Ethiopia, where patients generally demonstrate better adherence [31]. The study revealed that some participants had difficulties fasting and experienced medication shortages due to instability. Similar findings reported in prior studies, , which

Response: Thank you for the comment. We have corrected the issue by removing the unnecessary commas.

7. PLOS authors have the option to publish the peer review history of their article (what does this mean?). If published, this will include your full peer review and any attached files.If you choose “no”, your identity will remain anonymous but your review may still be made public.

Do you want your identity to be public for this peer review? For information about this choice, including consent withdrawal, please see our Privacy Policy.

Reviewer #2: No

[NOTE: If reviewer comments were submitted as an attachment file, they will be attached to this email and accessible via the submission site. Please log into your account, locate the manuscript record, and check for the action link "View Attachments". If this link does not appear, there are no attachment files.]

While revising your submission, please upload your figure files to the Preflight Analysis and Conversion Engine (PACE) digital diagnostic tool, https://pacev2.apexcovantage.com/. PACE helps ensure that figures meet PLOS requirements. To use PACE, you must first register as a user. Registration is free. Then, login and navigate to the UPLOAD tab, where you will find detailed instructions on how to use the tool. If you encounter any issues or have any questions when using PACE, please email PLOS at figures@plos.org. Please note that Supporting Information files do not need this step.

# Peer review: Decision Letter 2

**DOI:** 10.1371/journal.pone.0316505.r005

**Authors:** Ammal Metwally, Ammal Metwally, Ammal Metwally

19 Jan 2026

PONE-D-24-55247R2

Lived Experiences of Diabetes Self-Management in North Shoa, Ethiopia: A Phenomenological Inquiry

PLOS One

Dear Dr. Eshete,

Thank you for submitting your manuscript to PLOS ONE. After careful consideration, we feel that it has merit but does not fully meet PLOS ONE’s publication criteria as it currently stands. Therefore, we invite you to submit a revised version of the manuscript that addresses the points raised during the review process.

Please submit your revised manuscript by Mar 05 2026 11:59PM. If you will need more time than this to complete your revisions, please reply to this message or contact the journal office at plosone@plos.org. When you're ready to submit your revision, log on to https://www.editorialmanager.com/pone/ and select the 'Submissions Needing Revision' folder to locate your manuscript file.

Please include the following items when submitting your revised manuscript:

- A letter that responds to each point raised by the academic editor and reviewer(s). You should upload this letter as a separate file labeled 'Response to Reviewers'.
- A marked-up copy of your manuscript that highlights changes made to the original version. You should upload this as a separate file labeled 'Revised Manuscript with Track Changes'.
- An unmarked version of your revised paper without tracked changes. You should upload this as a separate file labeled 'Manuscript'.

-->If you would like to make changes to your financial disclosure, please include your updated statement in your cover letter. Guidelines for resubmitting your figure files are available below the reviewer comments at the end of this letter.

If applicable, we recommend that you deposit your laboratory protocols in protocols.io to enhance the reproducibility of your results. Protocols.io assigns your protocol its own identifier (DOI) so that it can be cited independently in the future. For instructions see: https://journals.plos.org/plosone/s/submission-guidelines#loc-laboratory-protocols. Additionally, PLOS ONE offers an option for publishing peer-reviewed Lab Protocol articles, which describe protocols hosted on protocols.io. Read more information on sharing protocols at https://plos.org/protocols?utm_medium=editorial-email&utm_source=authorletters&utm_campaign=protocols.

We look forward to receiving your revised manuscript.

Kind regards,

Ammal Mokhtar Metwally, Ph.D (MD)

Academic Editor

PLOS One

Journal Requirements:

If the reviewer comments include a recommendation to cite specific previously published works, please review and evaluate these publications to determine whether they are relevant and should be cited. There is no requirement to cite these works unless the editor has indicated otherwise.

Please review your reference list to ensure that it is complete and correct. If you have cited papers that have been retracted, please include the rationale for doing so in the manuscript text, or remove these references and replace them with relevant current references. Any changes to the reference list should be mentioned in the rebuttal letter that accompanies your revised manuscript. If you need to cite a retracted article, indicate the article’s retracted status in the References list and also include a citation and full reference for the retraction notice.

[Note: HTML markup is below. Please do not edit.]

Reviewers' comments:

Reviewer's Responses to Questions

**Comments to the Author**

1. If the authors have adequately addressed your comments raised in a previous round of review and you feel that this manuscript is now acceptable for publication, you may indicate that here to bypass the “Comments to the Author” section, enter your conflict of interest statement in the “Confidential to Editor” section, and submit your "Accept" recommendation.

Reviewer #2: (No Response)

**********

2. Is the manuscript technically sound, and do the data support the conclusions?

The manuscript must describe a technically sound piece of scientific research with data that supports the conclusions. Experiments must have been conducted rigorously, with appropriate controls, replication, and sample sizes. The conclusions must be drawn appropriately based on the data presented.

Reviewer #2: Yes

**********

3. Has the statistical analysis been performed appropriately and rigorously?

Reviewer #2: Yes

**********

4. Have the authors made all data underlying the findings in their manuscript fully available?

The PLOS Data policy requires authors to make all data underlying the findings described in their manuscript fully available without restriction, with rare exception (please refer to the Data Availability Statement in the manuscript PDF file). The data should be provided as part of the manuscript or its supporting information, or deposited to a public repository. For example, in addition to summary statistics, the data points behind means, medians and variance measures should be available. If there are restrictions on publicly sharing data—e.g. participant privacy or use of data from a third party—those must be specified.

Reviewer #2: Yes

**********

5. Is the manuscript presented in an intelligible fashion and written in standard English?

PLOS ONE does not copyedit accepted manuscripts, so the language in submitted articles must be clear, correct, and unambiguous. Any typographical or grammatical errors should be corrected at revision, so please note any specific errors here.

Reviewer #2: Yes

**********

6. Review Comments to the Author

Please use the space provided to explain your answers to the questions above. You may also include additional comments for the author, including concerns about dual publication, research ethics, or publication ethics. (Please upload your review as an attachment if it exceeds 20,000 characters)

Reviewer #2: I find this to be a well-conducted qualitative study addressing an important topic. The comments below are minor in nature and would not preclude advancement of the manuscript.

On page 11 of the abstract, the heading should read “Results” rather than “Result.” Additionally, the sentence “Although most of the patients followed their medications” is unclear and would benefit from more precise wording, such as clarifying whether patients took their medications as prescribed or followed medication instructions, if either of these statements is the intended meaning.

On page 14, the description of the interview guide and data collection process is thorough and well written; however, clarification is needed regarding what is meant by the interview guide being pretested in a “similar setting.” Specifying whether this refers to a comparable healthcare facility, patient population, or geographic context would strengthen methodological transparency.

On page 15, the researcher reflexivity statement is appropriate and clearly articulated, but the reference to the research team’s prior understanding of patients’ self-care practices from previous work should be supported with relevant citations to strengthen rigor and credibility.

In the discussion section (pages 25–30), several findings are individually compared with the same external Ethiopian study. For conciseness and improved readability, these comparisons could be consolidated by grouping related findings rather than repeatedly referencing the same study across multiple points.

Overall, these comments relate primarily to clarity, wording, citation, and organization and are minor in scope; they would not prevent the manuscript from advancing if approved by the remaining editorial committee.

**********

7. PLOS authors have the option to publish the peer review history of their article (what does this mean?). If published, this will include your full peer review and any attached files.

If you choose “no”, your identity will remain anonymous but your review may still be made public.

**Do you want your identity to be public for this peer review?**  For information about this choice, including consent withdrawal, please see our Privacy Policy.

Reviewer #2: No

**********

[NOTE: If reviewer comments were submitted as an attachment file, they will be attached to this email and accessible via the submission site. Please log into your account, locate the manuscript record, and check for the action link "View Attachments". If this link does not appear, there are no attachment files.]

To ensure your figures meet our technical requirements, please review our figure guidelines: https://journals.plos.org/plosone/s/figures

You may also use PLOS’s free figure tool, NAAS, to help you prepare publication quality figures: https://journals.plos.org/plosone/s/figures#loc-tools-for-figure-preparation.

NAAS will assess whether your figures meet our technical requirements by comparing each figure against our figure specifications.

# Author comment: Author response to Decision Letter 3

**DOI:** 10.1371/journal.pone.0316505.r006

18 Feb 2026

Dear Editorial Board Member!

PLOS ONE

Response to Editor Comments

I am happy to resubmit for revised version of “Lived Experiences of Diabetes Self-Management in North Shoa, Ethiopia: A Phenomenological Inquiry.” We have addressed your concerns point by point.

Journal Requirements:

If the reviewer comments include a recommendation to cite specific previously published works, please review and evaluate these publications to determine whether they are relevant and should be cited. There is no requirement to cite these works unless the editor has indicated otherwise.

Please review your reference list to ensure that it is complete and correct. If you have cited papers that have been retracted, please include the rationale for doing so in the manuscript text, or remove these references and replace them with relevant current references. Any changes to the reference list should be mentioned in the rebuttal letter that accompanies your revised manuscript. If you need to cite a retracted article, indicate the article’s retracted status in the References list and also include a citation and full reference for the retraction notice.

Reviewer's Responses to Questions

Comments to the Author

1. If the authors have adequately addressed your comments raised in a previous round of review and you feel that this manuscript is now acceptable for publication, you may indicate that here to bypass the “Comments to the Author” section, enter your conflict of interest statement in the “Confidential to Editor” section, and submit your "Accept" recommendation.

Reviewer #2: (No Response)

2. Is the manuscript technically sound, and do the data support the conclusions?

The manuscript must describe a technically sound piece of scientific research with data that supports the conclusions. Experiments must have been conducted rigorously, with appropriate controls, replication, and sample sizes. The conclusions must be drawn appropriately based on the data presented.

Reviewer #2: Yes

3. Has the statistical analysis been performed appropriately and rigorously?

Reviewer #2: Yes

4. Have the authors made all data underlying the findings in their manuscript fully available?

The PLOS Data policy requires authors to make all data underlying the findings described in their manuscript fully available without restriction, with rare exception (please refer to the Data Availability Statement in the manuscript PDF file). The data should be provided as part of the manuscript or its supporting information, or deposited to a public repository. For example, in addition to summary statistics, the data points behind means, medians and variance measures should be available. If there are restrictions on publicly sharing data—e.g. participant privacy or use of data from a third party—those must be specified.

Reviewer #2: Yes

5. Is the manuscript presented in an intelligible fashion and written in standard English?

PLOS ONE does not copyedit accepted manuscripts, so the language in submitted articles must be clear, correct, and unambiguous. Any typographical or grammatical errors should be corrected at revision, so please note any specific errors here.

Reviewer #2: Yes

6. Review Comments to the Author

Please use the space provided to explain your answers to the questions above. You may also include additional comments for the author, including concerns about dual publication, research ethics, or publication ethics. (Please upload your review as an attachment if it exceeds 20,000 characters)

Reviewer #2: I find this to be a well-conducted qualitative study addressing an important topic. The comments below are minor in nature and would not preclude advancement of the manuscript.

1. On page 11 of the abstract, the heading should read “Results” rather than “Result.” Additionally, the sentence “Although most of the patients followed their medications” is unclear and would benefit from more precise wording, such as clarifying whether patients took their medications as prescribed or followed medication instructions, if either of these statements is the intended meaning.

Response: thank you very much. We addressed based on the comments. We modified like, Despite high adherence to prescribed medications among most patients, many demonstrated inadequate practices in self-blood glucose monitoring, dietary practice, regular physical activity, and regular foot care.

2. On page 14, the description of the interview guide and data collection process is thorough and well written; however, clarification is needed regarding what is meant by the interview guide being pretested in a “similar setting.” Specifying whether this refers to a comparable healthcare facility, patient population, or geographic context would strengthen methodological transparency.

Response: thank you for this valuable comment. We have clarified that the interview guide was pretested in a comparable healthcare facility with a similar patient population and service context to the study setting, but outside the actual study sites. This clarification has been added to the Data Collection section to improve methodological transparency.

On page 15, the researcher reflexivity statement is appropriate and clearly articulated, but the reference to the research team’s prior understanding of patients’ self-care practices from previous work should be supported with relevant citations to strengthen rigor and credibility.

Response: thank you for this valuable comment. We have listed some of the the publication

• Effect of physical activity promotion program on adherence to physical exercise among patients with type II diabetes in North Shoa Zone Amhara region: a quasi-experimental study (https://pmc.ncbi.nlm.nih.gov/articles/PMC10114377/)

• Association of stress management behavior and diabetic self-care practice among diabetes type II patients in North Shoa Zone: a cross-sectional study. BMC Health Serv Res 23, 767 (2023). https://doi.org/10.1186/s12913-023-09752-6

• Effect of nutritional promotion intervention on dietary adherence among type II diabetes patients in North Shoa Zone Amhara Region: quasi-experimental study. J Health Popul Nutr 42, 49 (2023). https://doi.org/10.1186/s41043-023-00393-3

3. In the discussion section (pages 25–30), several findings are individually compared with the same external Ethiopian study. For conciseness and improved readability, these comparisons could be consolidated by grouping related findings rather than repeatedly referencing the same study across multiple points. Overall, these comments relate primarily to clarity, wording, citation, and organization and are minor in scope; they would not prevent the manuscript from advancing if approved by the remaining editorial committee.

Response: thank you for this valuable comment. The discussion section is organized under thematic subheadings, which may result in some repeated citations. This approach was intentionally used to ensure clarity and coherence within each theme. We kindly request that this justification be accepted.

7. PLOS authors have the option to publish the peer review history of their article (what does this mean?). If published, this will include your full peer review and any attached files.

If you choose “no”, your identity will remain anonymous but your review may still be made public.

Do you want your identity to be public for this peer review? For information about this choice, including consent withdrawal, please see our Privacy Policy.

Reviewer #2: No

[NOTE: If reviewer comments were submitted as an attachment file, they will be attached to this email and accessible via the submission site. Please log into your account, locate the manuscript record, and check for the action link "View Attachments". If this link does not appear, there are no attachment files.]

To ensure your figures meet our technical requirements, please review our figure guidelines: https://journals.plos.org/plosone/s/figures

You may also use PLOS’s free figure tool, NAAS, to help you prepare publication quality figures: https://journals.plos.org/plosone/s/figures#loc-tools-for-figure-preparation.

NAAS will assess whether your figures meet our technical requirements by comparing each figure against our figure specifications.

Attachment Submitted filename: Response to data publication consent and restrictions..pdf

# Peer review: Decision Letter 3

**DOI:** 10.1371/journal.pone.0316505.r007

**Authors:** Ammal Metwally, Ammal Metwally, Ammal Metwally, Ammal Metwally

13 Mar 2026

PONE-D-24-55247R3

Lived Experiences of Diabetes Self-Management in North Shoa, Ethiopia: A Phenomenological Inquiry

PLOS One

Dear Dr. Eshete,

Thank you for submitting your manuscript to PLOS ONE. After careful consideration, we feel that it has merit but does not fully meet PLOS ONE’s publication criteria as it currently stands. Therefore, we invite you to submit a revised version of the manuscript that addresses the points raised during the review process.

Please submit your revised manuscript by Apr 27 2026 11:59PM. If you will need more time than this to complete your revisions, please reply to this message or contact the journal office at plosone@plos.org. When you're ready to submit your revision, log on to https://www.editorialmanager.com/pone/ and select the 'Submissions Needing Revision' folder to locate your manuscript file.

Please include the following items when submitting your revised manuscript:

- A letter that responds to each point raised by the academic editor and reviewer(s). You should upload this letter as a separate file labeled 'Response to Reviewers'.
- A marked-up copy of your manuscript that highlights changes made to the original version. You should upload this as a separate file labeled 'Revised Manuscript with Track Changes'.
- An unmarked version of your revised paper without tracked changes. You should upload this as a separate file labeled 'Manuscript'.

-->If you would like to make changes to your financial disclosure, please include your updated statement in your cover letter. Guidelines for resubmitting your figure files are available below the reviewer comments at the end of this letter.

If applicable, we recommend that you deposit your laboratory protocols in protocols.io to enhance the reproducibility of your results. Protocols.io assigns your protocol its own identifier (DOI) so that it can be cited independently in the future. For instructions see: https://journals.plos.org/plosone/s/submission-guidelines#loc-laboratory-protocols. Additionally, PLOS ONE offers an option for publishing peer-reviewed Lab Protocol articles, which describe protocols hosted on protocols.io. Read more information on sharing protocols at https://plos.org/protocols?utm_medium=editorial-email&utm_source=authorletters&utm_campaign=protocols.

We look forward to receiving your revised manuscript.

Kind regards,

Ammal Mokhtar Metwally, Ph.D (MD)

Academic Editor

PLOS One

Journal Requirements:

If the reviewer comments include a recommendation to cite specific previously published works, please review and evaluate these publications to determine whether they are relevant and should be cited. There is no requirement to cite these works unless the editor has indicated otherwise.

Please review your reference list to ensure that it is complete and correct. If you have cited papers that have been retracted, please include the rationale for doing so in the manuscript text, or remove these references and replace them with relevant current references. Any changes to the reference list should be mentioned in the rebuttal letter that accompanies your revised manuscript. If you need to cite a retracted article, indicate the article’s retracted status in the References list and also include a citation and full reference for the retraction notice.

Additional Editor Comments:

Thank you for submitting the revised version of your manuscript and for your careful attention to the reviewers’ comments. The reviewers’ assessments are favorable, and I agree that the manuscript addresses an important topic and has merit. The study is well-positioned for publication, and I appreciate the effort invested in strengthening the work through revision.

At the same time, before the manuscript can be accepted in final form, a small number of editorial and reporting issues still need to be addressed to ensure consistency, clarity, and compliance with journal requirements. These issues do not call into question the overall suitability of the study for publication, but they should be corrected before final acceptance.

The most important point concerns the Data Availability Statement, which appears to require further clarification and internal consistency. The current submission materials suggest that all relevant data are fully available within the manuscript and supporting files, while other parts of the manuscript indicate that the interview transcripts are available only on reasonable request and under restricted conditions because they contain potentially sensitive information. Please revise this statement so that it is fully consistent across the manuscript and aligned with the journal’s data-sharing policy.

In addition, the manuscript would benefit from a final editorial clean-up. There are still minor revision artifacts and small formatting issues that should be removed. These include residual formatting text, duplicated or awkwardly merged wording in the Methods, and a few minor language and punctuation issues in the Abstract. While these are not substantive scientific concerns, they should be corrected to ensure the manuscript is ready for publication.

Please address the following points in your next revision:

Revise the Data Availability Statement to ensure that it accurately reflects the status of the qualitative data and is fully consistent throughout the submission files and manuscript text.

Remove artifacts that appear to have been carried over during editing.

Correct duplicated wording in the Methods section and any similarity elsewhere in the manuscript.

Perform a final language and typographical review, particularly in the Abstract, to correct minor punctuation, software naming, and sentence-level clarity issues.

Ensure that the final version is consistent and concise for all the manuscript sections

[Note: HTML markup is below. Please do not edit.]

Reviewers' comments:

Reviewer's Responses to Questions

**Comments to the Author**

1. If the authors have adequately addressed your comments raised in a previous round of review and you feel that this manuscript is now acceptable for publication, you may indicate that here to bypass the “Comments to the Author” section, enter your conflict of interest statement in the “Confidential to Editor” section, and submit your "Accept" recommendation.

Reviewer #2: All comments have been addressed

**********

2. Is the manuscript technically sound, and do the data support the conclusions?

The manuscript must describe a technically sound piece of scientific research with data that supports the conclusions. Experiments must have been conducted rigorously, with appropriate controls, replication, and sample sizes. The conclusions must be drawn appropriately based on the data presented.

Reviewer #2: Yes

**********

3. Has the statistical analysis been performed appropriately and rigorously?

Reviewer #2: Yes

**********

4. Have the authors made all data underlying the findings in their manuscript fully available?

The PLOS Data policy requires authors to make all data underlying the findings described in their manuscript fully available without restriction, with rare exception (please refer to the Data Availability Statement in the manuscript PDF file). The data should be provided as part of the manuscript or its supporting information, or deposited to a public repository. For example, in addition to summary statistics, the data points behind means, medians and variance measures should be available. If there are restrictions on publicly sharing data—e.g. participant privacy or use of data from a third party—those must be specified.

Reviewer #2: Yes

**********

5. Is the manuscript presented in an intelligible fashion and written in standard English?

PLOS ONE does not copyedit accepted manuscripts, so the language in submitted articles must be clear, correct, and unambiguous. Any typographical or grammatical errors should be corrected at revision, so please note any specific errors here.

Reviewer #2: Yes

**********

6. Review Comments to the Author

Please use the space provided to explain your answers to the questions above. You may also include additional comments for the author, including concerns about dual publication, research ethics, or publication ethics. (Please upload your review as an attachment if it exceeds 20,000 characters)

Reviewer #2: (No Response)

**********

7. PLOS authors have the option to publish the peer review history of their article (what does this mean?). If published, this will include your full peer review and any attached files.

If you choose “no”, your identity will remain anonymous but your review may still be made public.

**Do you want your identity to be public for this peer review?**  For information about this choice, including consent withdrawal, please see our Privacy Policy.

Reviewer #2: No

**********

[NOTE: If reviewer comments were submitted as an attachment file, they will be attached to this email and accessible via the submission site. Please log into your account, locate the manuscript record, and check for the action link "View Attachments". If this link does not appear, there are no attachment files.]

To ensure your figures meet our technical requirements, please review our figure guidelines: https://journals.plos.org/plosone/s/figures

You may also use PLOS’s free figure tool, NAAS, to help you prepare publication quality figures: https://journals.plos.org/plosone/s/figures#loc-tools-for-figure-preparation.

NAAS will assess whether your figures meet our technical requirements by comparing each figure against our figure specifications.

# Author comment: Author response to Decision Letter 4

**DOI:** 10.1371/journal.pone.0316505.r008

23 Apr 2026

Dear Editorial Board Member!

PLOS ONE

Response to Editor Comments

I am happy to resubmit for the revised version of “Lived Experiences of Diabetes Self-Management in North Shoa, Ethiopia: A Phenomenological Inquiry.” We have addressed your concerns point by point.

Comments

PONE-D-24-55247R3

Lived Experiences of Diabetes Self-Management in North Shoa, Ethiopia: A Phenomenological Inquiry

PLOS One

Please address the following points in your next revision:

1. Revise the Data Availability Statement to ensure that it accurately reflects the status of the qualitative data and is fully consistent throughout the submission files and manuscript text.

• Response: we addressed accordingly the comments and make similar at the submission files and manuscript text

2. Remove artifacts that appear to have been carried over during editing.

• Response: We have been carefully identified and removed to ensure clarity, consistency of the findings

3. Correct duplicated wording in the Methods section and any similarity elsewhere in the manuscript

• Response: We addressed the comments throughout the manuscript accordingly

4. Perform a final language and typographical review, particularly in the Abstract, to correct minor punctuation, software naming, and sentence-level clarity issues. Ensure that the final version is consistent and concise for all the manuscript sections

• Response: we addressed the comments throughout the manuscript accordingly

Attachment Submitted filename: Response to Non-author contact.pdf

# Peer review: Decision Letter 4

**DOI:** 10.1371/journal.pone.0316505.r009

**Authors:** Ammal Metwally, Ammal Metwally, Ammal Metwally, Ammal Metwally, Ammal Metwally

1 Jun 2026

Lived Experiences of Diabetes Self-Management in North Shoa, Ethiopia: A Phenomenological Inquiry

PONE-D-24-55247R4

Dear Dr. Eshete,

We’re pleased to inform you that your manuscript has been judged scientifically suitable for publication and will be formally accepted for publication once it meets all outstanding technical requirements.

Within one week, you’ll receive an e-mail detailing the required amendments. When these have been addressed, you’ll receive a formal acceptance letter and your manuscript will be scheduled for publication.

An invoice will be generated when your article is formally accepted. Please note, if your institution has a publishing partnership with PLOS and your article meets the relevant criteria, all or part of your publication costs will be covered. Please make sure your user information is up-to-date by logging into Editorial Manager at Editorial Manager® and clicking the ‘Update My Information' link at the top of the page. For questions related to billing, please contact billing support.

If your institution or institutions have a press office, please notify them about your upcoming paper to help maximize its impact. If they’ll be preparing press materials, please inform our press team as soon as possible -- no later than 48 hours after receiving the formal acceptance. Your manuscript will remain under strict press embargo until 2 pm Eastern Time on the date of publication. For more information, please contact onepress@plos.org.

Kind regards,

Ammal Mokhtar Metwally, Ph.D (MD)

Academic Editor

PLOS One

Additional Editor Comments (optional):

Reviewers' comments:

Reviewer's Responses to Questions

**Comments to the Author**

1. If the authors have adequately addressed your comments raised in a previous round of review and you feel that this manuscript is now acceptable for publication, you may indicate that here to bypass the “Comments to the Author” section, enter your conflict of interest statement in the “Confidential to Editor” section, and submit your "Accept" recommendation.

Reviewer #2: All comments have been addressed

**********

2. Is the manuscript technically sound, and do the data support the conclusions?

The manuscript must describe a technically sound piece of scientific research with data that supports the conclusions. Experiments must have been conducted rigorously, with appropriate controls, replication, and sample sizes. The conclusions must be drawn appropriately based on the data presented.

Reviewer #2: Yes

**********

3. Has the statistical analysis been performed appropriately and rigorously?

Reviewer #2: Yes

**********

4. Have the authors made all data underlying the findings in their manuscript fully available?

The PLOS Data policy requires authors to make all data underlying the findings described in their manuscript fully available without restriction, with rare exception (please refer to the Data Availability Statement in the manuscript PDF file). The data should be provided as part of the manuscript or its supporting information, or deposited to a public repository. For example, in addition to summary statistics, the data points behind means, medians and variance measures should be available. If there are restrictions on publicly sharing data—e.g. participant privacy or use of data from a third party—those must be specified.

Reviewer #2: Yes

**********

5. Is the manuscript presented in an intelligible fashion and written in standard English?

PLOS ONE does not copyedit accepted manuscripts, so the language in submitted articles must be clear, correct, and unambiguous. Any typographical or grammatical errors should be corrected at revision, so please note any specific errors here.

Reviewer #2: Yes

**********

6. Review Comments to the Author

Please use the space provided to explain your answers to the questions above. You may also include additional comments for the author, including concerns about dual publication, research ethics, or publication ethics. (Please upload your review as an attachment if it exceeds 20,000 characters)

Reviewer #2: All formerly submitted required questions have been answered and all responses meet formatting specifications.

**********

7. PLOS authors have the option to publish the peer review history of their article (what does this mean?). If published, this will include your full peer review and any attached files.

If you choose “no”, your identity will remain anonymous but your review may still be made public.

**Do you want your identity to be public for this peer review?**  For information about this choice, including consent withdrawal, please see our Privacy Policy.

Reviewer #2: No

**********

# Editor report: Acceptance letter

**DOI:** 10.1371/journal.pone.0316505.r010

**Authors:** Ammal Metwally, Ammal Metwally, Ammal Metwally, Ammal Metwally, Ammal Metwally

PONE-D-24-55247R4

PLOS One

Dear Dr. Eshete,

I'm pleased to inform you that your manuscript has been deemed suitable for publication in PLOS One. Congratulations! Your manuscript is now being handed over to our production team.

At this stage, our production department will prepare your paper for publication. This includes ensuring the following:

* All references, tables, and figures are properly cited

* All relevant supporting information is included in the manuscript submission,

* There are no issues that prevent the paper from being properly typeset

You will receive further instructions from the production team, including instructions on how to review your proof when it is ready. Please keep in mind that we are working through a large volume of accepted articles, so please give us a few days to review your paper and let you know the next and final steps.

Lastly, if your institution or institutions have a press office, please let them know about your upcoming paper now to help maximize its impact. If they'll be preparing press materials, please inform our press team within the next 48 hours. Your manuscript will remain under strict press embargo until 2 pm Eastern Time on the date of publication. For more information, please contact onepress@plos.org.

You will receive an invoice from PLOS for your publication fee after your manuscript has reached the completed accept phase. If you receive an email requesting payment before acceptance or for any other service, this may be a phishing scheme. Learn how to identify phishing emails and protect your accounts at https://explore.plos.org/phishing.

If we can help with anything else, please email us at customercare@plos.org.

Thank you for submitting your work to PLOS ONE and supporting open access.

Kind regards,

PLOS ONE Editorial Office Staff

on behalf of

Professor Ammal Mokhtar Metwally

Academic Editor

PLOS One
